# Population genomics of rapid evolution in natural populations: polygenic selection in response to power station thermal effluents

**DOI:** 10.1186/s12862-019-1392-5

**Published:** 2019-02-26

**Authors:** David I. Dayan, Xiao Du, Tara Z. Baris, Dominique N. Wagner, Douglas L. Crawford, Marjorie F. Oleksiak

**Affiliations:** Rosenstiel School of Marine and Atmospheric Science, University of Miami, 4600 Rickenbacker Causeway, Miami, FL 33149 USA

**Keywords:** Population genomics - empirical, Selective sweeps, Polygenic adaptation, Adaptation, Fish

## Abstract

**Background:**

Examples of rapid evolution are common in nature but difficult to account for with the standard population genetic model of adaptation. Instead, selection from the standing genetic variation permits rapid adaptation via soft sweeps or polygenic adaptation. Empirical evidence of this process in nature is currently limited but accumulating.

**Results:**

We provide genome-wide analyses of rapid evolution in *Fundulus heteroclitus* populations subjected to recently elevated temperatures due to coastal power station thermal effluents using 5449 SNPs across two effluent-affected and four reference populations. Bayesian and multivariate analyses of population genomic structure reveal a substantial portion of genetic variation that is most parsimoniously explained by selection at the site of thermal effluents. An F_ST_ outlier approach in conjunction with additional conservative requirements identify significant allele frequency differentiation that exceeds neutral expectations among exposed and closely related reference populations. Genomic variation patterns near these candidate loci reveal that individuals living near thermal effluents have rapidly evolved from the standing genetic variation through small allele frequency changes at many loci in a pattern consistent with polygenic selection on the standing genetic variation.

**Conclusions:**

While the ultimate trajectory of selection in these populations is unknown and we survey only a minority of genomic loci, our findings suggest that polygenic models of adaptation may play important roles in large, natural populations experiencing recent selection due to environmental changes that cause broad physiological impacts.

**Electronic supplementary material:**

The online version of this article (10.1186/s12862-019-1392-5) contains supplementary material, which is available to authorized users.

## Background

Understanding the population genetic basis of local adaptation is one of the principal goals of evolutionary biology. Historically, theoretical models of adaptation have focused on selection on one or a few genetic loci and populations that are mutationally limited. Under these assumptions, a typical adaptive walk is a long process characterized by successive fixations of large effect alleles that arise via mutation after the onset of selection [1]. Fixation of each adaptive allele leads to a strong selective signature among genomic variants in linkage disequilibrium with the causative mutation, producing a “hard sweep” [2]. This model has provided a detailed set of predictions that form the basis of many empirical tests for selection [3, 4] and has been supported by population genetic examinations of candidate genes, where the genotype to phenotype map for known adaptive traits is well characterized [5, 6].

While this standard hard sweep model is often invoked to explain the genetic basis of adaptation among species that diverged millions of generations ago or populations that diverged thousands of generations ago, many of the most salient evolutionary questions today, and perhaps historically, occur on much more rapid time scales, e.g. adaptation to novel environments during species introductions [7] and in spatially restricted populations coping with global climate change [8]. Examples of rapid phenotypic evolution abound in both the laboratory and in nature and can occur over time scales as short as tens of generations [9]. Hard sweeps are unlikely to drive these rapid phenotypic shifts because there is insufficient time to overcome the lag period in the standard model; in this standard model, adaptation requires an adaptive mutation to appear and reach high enough frequency to escape stochastic loss. Instead of proceeding via hard sweeps, rapid evolution is proposed to proceed by either soft-sweeps or polygenic adaptation. Under the soft sweep model, selection sweeps adaptive alleles to high frequency, but these alleles are borne on multiple haplotypes, either because they arise independently or they are present in the standing genetic variation long enough to become unlinked from nearby variation [10, 11]. In the latter case, soft sweeps can facilitate rapid evolution because selection can act on alleles already segregating at moderate frequency at the onset of selection [12], avoiding the aforementioned lag period.

Soft sweeps appear to have driven local adaptation in a number of empirical examinations [13–16]. Yet, the traits under selection in such studies often demonstrate a simple, oligogenic genetic architecture. In contrast, natural selection is expected to operate primarily on highly integrated, complex performance traits [17] that are characterized by highly polygenic genetic architectures [18], i.e. quantitative traits. In the case of quantitative traits, genetic architecture may be so diffuse as to approximate Fisher’s infinitesimal model, which assumes an infinite number of loci each with an infinitely small effect [19], and polygenic adaptation may play an important role [20]. Multilocus simulations suggest that although sweeps at a minority of loci are possible, polygenic genetic architectures reduce the number of adaptive fixations that contribute to adaptation and that phenotypic evolution can proceed extremely rapidly via minor changes in allele frequency changes at many loci [21–23].

In the case of local adaptation, these predictions regarding the nature of adaptive genetic variants are additionally complicated by the influence of gene flow [24]. Gene flow can swamp locally advantageous alleles, leading to a bias towards large effect alleles that are more resistant to swamping [24], or tight linkage among multiple small effect loci that segregate in the populations as de facto large effect alleles [25]. This influence of gene flow leads to a tendency against small allele frequency changes at many loci and towards sweep scenarios, where adaptive allele frequencies vary greatly across populations [26]. Despite this predicted tendency for gene flow to bias the evolution of quantitative traits towards sweeps of large effect alleles, polygenic adaptation may play a significant role in the short run, because it takes time for “supergene” structural variants or similar effects to establish, e.g. [27, 28], or variants in low recombination areas of the genome that protect genomic regions containing adaptive variants from gene flow, e.g. [29], to enter the population and increase in frequency [30].

In this investigation, we seek to test these predictions regarding the nature of genetic variants contributing to rapid local adaptation in populations of the estuarine fish *Fundulus heteroclitus* exposed to the thermal effluents of coastal power stations. We sampled *F. heteroclitus* populations near the effluents of two power stations: Oyster Creek nuclear generating station in New Jersey and Brayton Point generating station in Massachusetts. Thermal effluents produced by coastal power stations provide a recent source of environmental variation that is both localized and well quantified. Effluents from both power plants have produced significant thermal impacts since the beginning of their operation in the late 1960s. The Oyster Creek thermal effluent is discharged along a modified river for approximately 3 km into the intracoastal Barnegat Bay. Temperatures range from 10 to 13 °C above ambient at the discharge site and 4–5 °C above ambient where Oyster Creek joins Barnegat Bay. Beyond this point, the effluent’s thermal influence is limited to a ~ 2 km radius from the mouth of Oyster Creek [31]. Documented ecological impacts of thermal input at Oyster Creek include maintenance of non-native, warm-adapted species not found elsewhere in the region [32] as well as decreased growth rates and failed spawning events in benthic molluscs [31]. At Brayton Point, effluent is released into the surrounding estuary, Mount Hope Bay, at 7–16 °C above ambient temperatures. This effluent leads to ~ 1 °C temperature anomaly throughout Mount Hope Bay [33], but has varied thermal impacts on smaller spatial scales due to incomplete mixing and advection of the thermal plume [34]. There are few predicted ecological impacts of the thermal effluent at Brayton Point [35, 36], although increased temperature may interact with other anthropogenic stressors in this region [37].

Power station thermal effects lead to thermal adaptation among exposed populations. Indeed, comparisons of natural populations living in or near thermal effluents have traditionally been used to parse adaptive genetic variation from neutral genetic variation. Allelic selection among electrophoretic variants of candidate loci is frequently reported in populations exposed to thermal effluents [38–40]. In *F. heteroclitus*, a northern thermal effluent population has allele frequencies more similar to distant, warm-adapted southern populations than more closely-related northern populations at several allozyme loci [41]. Selection due to thermal effluents can also elicit rapid phenotypic evolution. Largemouth bass (*Micropterus salmoides*) living in effluent ponds demonstrate increased frequency of more thermally stable isozymes after fifteen generations, and allele frequencies at these loci return to ancestral levels after just ten [42].

While the genetic architecture of thermal adaptation is not well known [43], we expect thermal adaptation to have a highly polygenic basis, in contrast with other recent population genomic studies of rapid evolution, such as rapid evolution of resistance to pesticides. Selection in response to a single or related chemicals is often mediated through a single pathway or even a single genetic locus [44–46], and this specificity lends itself to a narrow genomic basis of adaptation [47]. Temperature broadly impacts all physiological systems through its effects on biochemical reaction rates and biomolecular structures [48, 49]. Additionally, thermal variation has indirect impacts mediated through more complex ecological changes, e.g. species interactions [50], further increasing the number of genetic variants that might contribute to fitness in an altered thermal environment. Accordingly, rapid thermal adaptation is likely to have a different genomic basis than other well-understood examples of rapid evolution because the selection target is expected to be so broad.

Our investigation extends early allozyme data by directly examining variation at thousands of genotyping-by-sequencing derived genetic markers. We provide population genomic, functional genomic and phenotypic evidence suggestive of adaptation in natural *Fundulus heteroclitus* populations exposed to thermal effluents. We then consider the genomic variation near candidate adaptive loci in light of recent theory regarding the nature of genetic variants contributing to local adaptation. Specifically, our data sheds light on the nature of adaptive variants when (i) selection is very recent, (ii) the selective environment is spatially restricted with respect to the extent of historical gene flow among populations, (iii) the effective population size is large, and (iv) the traits under selection are likely to have a highly polygenic basis. Under these restrictions we do not expect hard sweeps to play a significant role, and theory suggests that adaptation might occur via soft sweeps or polygenic adaptation.

## Results

### Samples and filtering

**Fig. 1:**
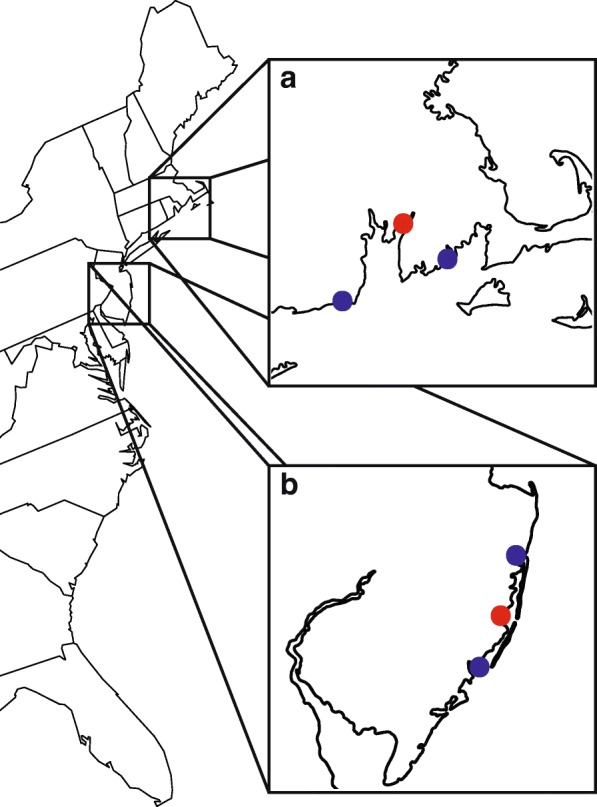
Sampling locations and triad design. Each thermal effluent population (red markers) is surrounded by two reference populations (blue markers). The northern triad (**a**) is the Brayton Point generating station population and its references. The southern triad (**b**) consists of the Oyster Creek nuclear generating station population and its references. Map data are drawn from US Department of Census and visualized using the *maps* package in R

After filtering on the basis of depth, missingness, minor allele frequency and Hardy-Weinberg equilibrium our final single nucleotide polymorphism (SNP) dataset consisted of 5449 SNPs among 239 individuals from six populations. *F. heteroclitus* populations were sampled in two “triads” [51], each consisting of a single thermal effluent (TE) site bordered on either side along the coast by a reference site (Fig. 1). The two TE populations are Oyster Creek and Brayton Point (Table 1). Mean read depth per SNP per individual was 26.29 ± 0.43 for the full SNP dataset (Additional file 1: Figure S1).

**Table 1:** Pairwise estimates of nucleotide diversity and population differentiation among experimental populations

	N	RB	OC	Mg	SR	BP	HB
RB	40	**0.1518**	0.0095	0.0081	0.1089	0.1256	0.1107
OC	36	0.1198	**0.0973**	0.0104	0.107	0.1261	0.1095
Mg	37	0.1483	0.1184	**0.1479**	0.0891	0.1063	0.0908
SR	41	0.1402	0.1111	0.1353	**0.1055**	0.0252	0.0318
BP	47	0.1418	0.1125	0.1367	0.1048	**0.1026**	0.0272
HB	38	0.138	0.1093	0.1331	0.1048	0.1028	**0.1015**

Values above the diagonal are mean pairwise F_ST_ values across all 5.4 k SNPs. Values along the diagonal (bold) are mean nucleotide diversity (π) within populations. Values below the diagonal are mean proportion of pairwise differences (π). Population abbreviations: Oyster Creek Triad (Southern Reference – Rutgers Basin (RB), TE Population – Oyster Creek Generating Station (OC), Northern Reference – Mantoloking, New Jersey (Mg)) Brayton Point Triad (Southern Reference – Succotash Marsh, Matunuck, Rhode Island (SR), TE Population – Brayton Point Generating Station (BP), Northern Reference – Horseneck Beach, Massachusetts (HB)). N: number of individuals in final SNP dataset

### Genome-wide diversity estimates and neutral population genetic structure

We identified substantial genetic diversity both within and among populations (Tables 1 and 2). Mean estimated pairwise F_ST_ values using the full SNP dataset ranged from 0.008–0.126 across the populations. Mean genetic diversity (π) estimates ranged from 0.103–0.148 among populations and 0.097–0.152 within each population (Table 1). Note, however, that these π estimates are inflated relative to true genome-wide averages because they are calculated using only SNPs from polymorphic sequence tags used to generate our SNP dataset, instead these data should be used to compare among populations in this study.

**Table 2:** AMOVA Results

Variance Component	df	% variation	Φ Statistic	P
Among triads	1	9.59	Φ_CT_ = 0.09593	*p* = 0.099
Among populations within triads	4	0.69	Φ_SC_ = 0.00762*	*p* < 0.00001
Among individuals within populations	472	89.72	Φ_ST_ = 0.10282*	p < 0.00001
Total	477			

Analysis of molecular variance (AMOVA) on the full SNP dataset partitioned 9.6% of the total genotypic variation to differences among triads, 0.7% among populations within a triad and the remainder (89.7%) within each population. The variance component among triads in the AMOVA was not significant while the other variance components were significant, suggesting that although the portion of genetic variation among triads is large, much of this variation is attributable to among populations structure; i.e.*,* the larger grouping (triads) is an artificial product of the hierarchical population sampling we employed. AMOVA conducted on a “neutral” SNP dataset that excluded outliers from any pairwise comparison within a triad produced qualitatively similar results, with the exception that the extent of among triad variation was reduced (Additional file 2: Table S1).

**Fig. 2:**
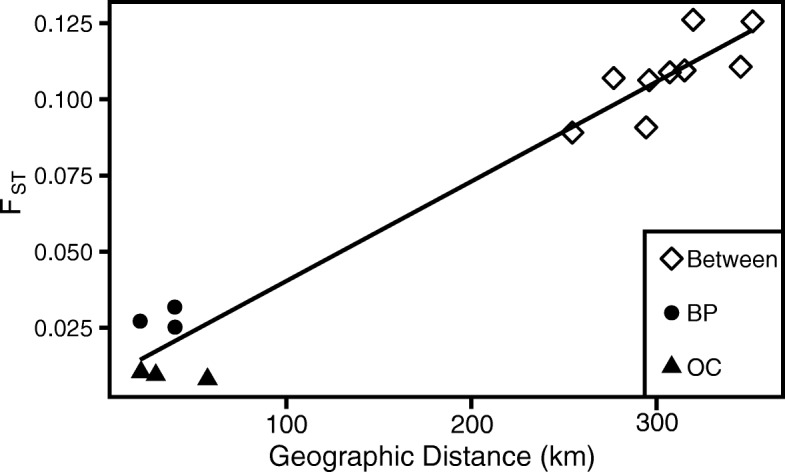
Isolation by distance. Geographic distance along the coast vs. genetic distance as estimated by mean genome-wide F_ST_ value for comparisons within the Brayton Point (BP, filled circles) and Oyster Creek (OC, filled triangles) triads and comparisons between triads (open diamonds). There is significant isolation by distance for all comparisons (p < 0.01, Mantel test, 9999 simulations), but not within triads (*p* > 0.5, Mantel test, 9999 simulations)

We examined populations within each triad for isolation by distance (IBD) using Mantel Tests (Fig. 2) [52]. While there is significant IBD across all populations among triads (*p* < 0.01, Mantel test of pairwise F_ST_ based on all loci vs. pairwise geographic distance, 9999 simulations), neither triad showed significant IBD among its populations (Mantel test, *p* > 0.5, 9999 simulations), nor is there a trend towards IBD using genome wide F_ST_ (Fig. 2). Therefore, the demographic assumptions inherent to outlier analyses are strongly violated when comparisons drawn across triads but valid within triads.

STRUCTURE identified no genetic substructure within a triad when all 5449 SNPs were used, i.e. best *k* was 1. Similarly, no structure was observed when using a putatively neutral set of SNPs created by excluding outliers. STRUCTURE detected population structure within the Brayton Point triad after thinning the full dataset for SNPs in linkage disquilibrium (r^2^ > 0.5) (Additional file 3: Figure S8). The best K was 3, with each population dominated by a single ancestry cluster (Additional file 4: Figure S3). No similar sensitivity to linked SNPs was found in the Oyster Creek triad. Best k was 1 and there was no large difference in cluster membership among the three populations (Additional file 4: Figure S3).

### Outlier analyses and candidate loci

**Fig. 3:**
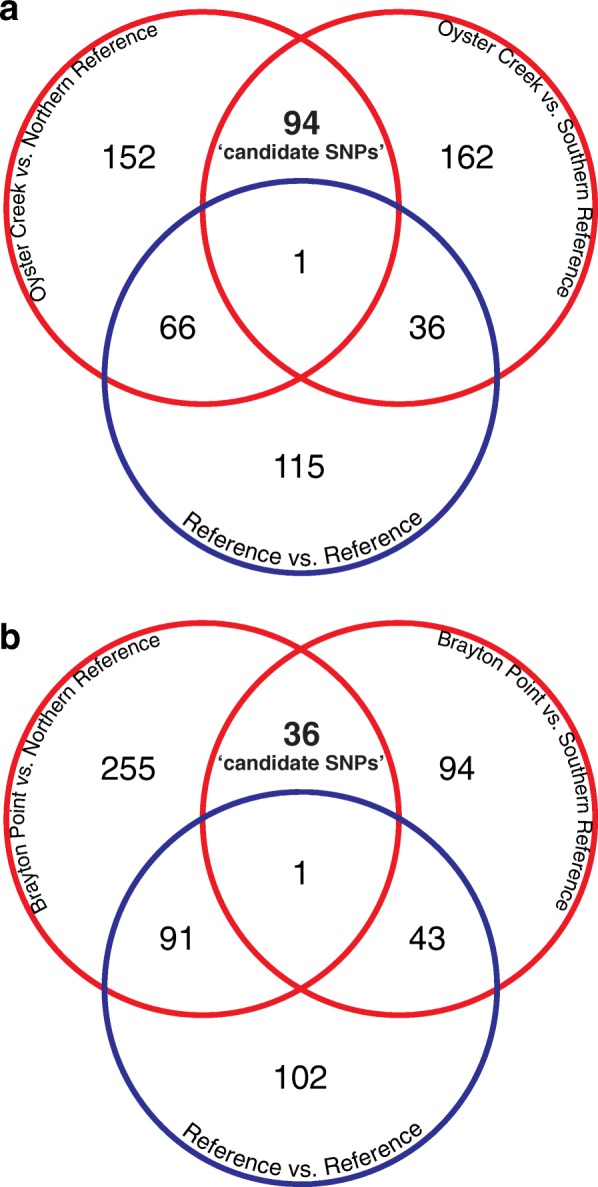
Candidates. Number of significant outliers in pairwise comparisons of thermal effluent (TE) populations vs. reference populations (red circles) and pairwise comparisons of reference vs. reference populations (blue circles) for (**a**) the Brayton Point (BP) and (**b**) the Oyster Creek (OC) (**b**) triads. Candidate loci for each triad (‘candidate’) are those that are significant outliers in both TE vs. reference comparisons (red circles), but not in the reference vs. reference comparison (blue)

To distinguish loci that have neutral divergence patterns from loci that may be evolving by natural selection among populations within a triad, we conducted outlier analyses using the FDIST2 algorithm implemented in Lositan [53]. An outlier analysis uses empirical data to simulate a neutral distribution of F_ST_ values for a given level of expected heterozygosity. SNPs with F_ST_ values that significantly exceed the simulated distribution with a modified FDR of 5% [54, 55] were considered outliers. The numbers of significant outliers in any single pairwise comparison among populations ranged from 3.2 to 5.7% of the total SNPs (Fig. 3) and were distributed across the observed heterozygosity range (Additional file 5: Figure S2). Within the Oyster Creek triad, 624 SNPs were identified as outlier loci in any of the three pairwise comparisons; 619 were identified in the Brayton Point triad (Fig. 3).

We identified potentially adaptive SNPs among these outliers using the triad experimental design. Specifically, we refer to SNPs that were identified as outliers in both pairwise comparisons of TE vs. reference populations, but not in the reference vs. reference comparison as candidate loci (see Fig. 3) [51] – see Discussion for details about this evolutionary inference. This approach revealed 94 candidate loci in the Oyster Creek TE population and 36 candidate loci in the Brayton Point TE population where population differentiation may be due to selection unique to the effluent site. The candidate loci for each triad are significantly enriched for SNPs with reduced nucleotide diversity (θ_π_) and Tajima’s D (Table 3).

**Table 3:** Nucleotide Diversity and Allele Frequency Spectrum at Candidate Loci vs. Genome-wide

Triad	SNP set	θ_π_	Tajima’s D
Brayton Point	Candidate Loci All Loci	0.0524*** 0.0873	−0.831**** − 0.599
Oyster Creek	Candidate Loci All Loci	0.0693**** 0.1262	−0.769**** − 0.431

Median values of within triad pairwise nucleotide divergence (θ_π_), and Tajima’s D for the triad candidate SNPs vs all SNPs. Asterisks denote significance level of one-tailed Wilcoxon rank sum test (*: *p* < 0.05, **: *p* < 0.01, ***: *p* < 0.001, ****: *p* < 0.0001)

### Inferring selection from population genetic structure

**Fig. 4:**
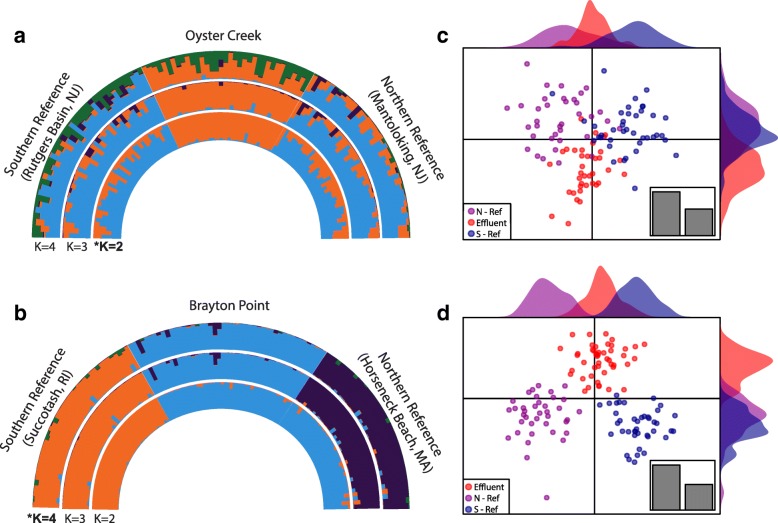
Population genetic structure within triads. STRUCTURE plots for Oyster Creek (**a**) and Brayton Point (**b**) triads based on data from the most differentiated loci (all pairwise outliers within a triad). Each individual is represented with a radial line that is partitioned into colors according to modeled admixture proportions for *k* ancestral populations. Results for *k =* 2–4 are presented with best *k* denoted by an asterisk. DAPC plots for Oyster Creek (**c**) and Brayton Point (**d**) triads using the full SNP dataset. Each individual’s position along the first two principal components (discriminant functions) is shown with a point, with populations identified by color. The relative eigenvalues of the first (horizontal) and second (vertical) principal components are shown in the bar plot at the bottom right of each figure

Genetic structure potentially resulting from varying selection among populations within a triad was inferred using two methods: a model-based Bayesian approach (STRUCTURE) using the most differentiated loci and a non-model-based multivariate approach (discriminant analysis of principal components (DAPC)) using the full SNP dataset (Fig. 4, Additional file 4: Figure S3). Given a number of ancestral populations or genetic clusters (*k*), STRUCTURE estimates the probability that an individual derives its ancestry from a particular genetic cluster at the loci provided. DAPC maximizes among population differences while minimizing within group variation by combining principal components of genetic variation.

#### Structure

First, we ran STRUCTURE using only SNPs that demonstrated significant differentiation among populations. This STRUCTURE analysis used the union of outlier SNPs from all three pairwise comparisons within a triad (624 SNPs for the Oyster Creek triad and 619 for the Brayton Point triad) at a range of putative population clusters between 1 and 6. Thus these results do not reflect population genetic structure genome-wide (see above for these results); rather they reflect differences among the populations at the most differentiated loci. These STRUCTURE analyses produced different results between the two triads. For the Oyster Creek triad, *K* = 2 captured most of the structure among the populations at the most differentiated loci (Fig. 4a, see methods for details). Using two population clusters, the two reference populations group with one another, separate from the TE population. While there are admixed individuals in all populations, the two reference populations were dominated by a single cluster while the TE population was dominated by a second cluster. Group membership in the first cluster was 75 and 68% for the two reference populations and 5% for Oyster Creek (TE). Results at increasing *K* values are qualitatively similar; the identity of a population as reference or effluent-affected predicts the major inferred ancestry cluster among the Oyster Creek triad populations. For the Brayton Point triad, *K* = 3 explains most of the structure among populations (Fig. 4b, see methods for details). At *K* = 3, each population is dominated by its own cluster, such that each population is unique. These results are robust to linkage disequilibrium (LD) among differentiated SNPs. Submitting an LD-thinned set of outlier SNPs to STRUCTURE produces qualitatively similar results (Additional file 6: Figure S9).

For both the Oyster Creek and Brayton Point triads we conducted an additional analysis. The most differentiated loci among populations within a triad are not representative of the genome-wide site frequency spectrum. Pairwise outliers are enriched for among loci with lower minor allele frequency (one tailed Wilcoxon rank sum test *p* = 0.038, and *p* = 2.2 × 10^− 16^, for BP and OC respectively). To investigate the possibility that the population genetic structure described above varies from structure at loci in the neutral SNP dataset because of differences in site frequency spectrum rather than high differentiation, e.g. STRUCTURE is more sensitive when using more or less common alleles, we bootstrap sampled the neutral dataset to match the outlier dataset analyzed above with respect to minor allele frequency. This site frequency spectrum matched neutral dataset did not demonstrate any evidence of structure within a triad suggesting that the structure identified using differentiated loci is not an artifact arising from the use of STRUCTURE on datasets that varied in their site frequency spectra.

#### Discriminant analysis of principal components

We also conducted discriminant analysis of principal components (DAPC) for the triads [56]. In contrast to the STRUCTURE results, DAPC performed on the full SNP dataset identified similar patterns of population structure for both triads. The first discriminant function in DAPC represents the major axis of genetic structure among populations. The three populations were distributed along this primary axis of genetic variation in a pattern consistent spatial autocorrelation; the TE population is intermediate to the two references (Fig. 4d). The second major axis of genetic variation used to discriminate between the populations explained less variation (eigenvalues Brayton Point: 297.8 vs. 169.6; eigenvalues Oyster Creek: 84.4 vs. 51.2) and revealed a population genetic structure pattern that is not consistent with their geographic distribution. Along the second major axis of genetic variation the two reference populations demonstrated substantial overlap while the TE population is distinct. Thus, for both triads the second major axis of genetic differences among populations for all 5449 loci in the SNP dataset does not fit the neutral expectation: the two reference populations are more similar to each other than either is to the TE population.

#### Environmental association - redundancy analysis

Finally, we used two multivariate approaches, redundancy analysis (RDA) and partial redundancy analysis (pRDA) [57] to examine the extent to which spatial variables and the presence of effluents could be used to explain patterns in the genetic variation among populations (Table 4). For Oyster Creek, the first 51 principal components of account for 53% of the total genetic variation. Only the first distance based Moran’s eigenvector map (dbMEM) had significant Moran’s I. The RDA was significant (*p* = 0.001, 1000 permutations). Variance partitioning revealed that 0.51% of the total genetic variation was attributable to spatial variation alone (27.9% of F_ST_), 0.49% attributable to effluents (26.8% of F_ST_) and no variation explained jointly (Table 4). The total constrained variance accounted for 54.6% of F_ST_. pRDA demonstrated that effluents and spatial distance each significantly explained genetic variation once controlling for the other (*p* = 0.001, 1000 permutations) (Table 4).

**Table 4:** RDA results for both triads considered jointly and each triad separately

Dataset	Percent Total Constrained Variance	F_ST_ Explained	Explanatory Variables	PVE	F_ST_ Explained per variable
Both Triads	0.77%	6.5%	dbMEM-1***	0.40%	3.4%
			Effluents***	0.36%	3.1%
Oyster Creek	0.99%	54.6%	dbMEM-1***	0.51%	27.9%
			Effluents***	0.49%	26.8%
Brayton Point	1.73%	27.3%	dbMEM-1***	0.86%	13.5%
			Effluents***	0.89%	13.9%

Asterisks denote significance of the variable in pRDA after conditioning the genetic data on all other variables, PTCV is percent total constrained variance among the major principal components of genetic variation for the full model, F_ST_ explained is the portion of among population genetic differentiation (F_ST_) explained by the total constrained variance weighted by the variance retained in the major principal components of genetic variation, PVE is percent of variance that is explained by each of the explanatory variable according to variance partitioning. Significant explanatory variables are indicated with the following symbols: ***P = 0.001

For Brayton Point the first 55 principal components of account for 44% of the total genetic variation. Only the first dbMEM had significant Moran’s I. The RDA was significant (*p* = 0.001, 1000 permutations) with 0.86% of the genetic variation attributable to spatial variation alone (13.5% of F_ST_), 0.89% attributable to effluents (13.9% of F_ST_) and no variation explained jointly (Table 4). The total constrained variance accounted for 27.3% of F_ST_. pRDA demonstrated that effluents and spatial distance each significantly explained genetic variation once controlling for the other (*p* = 0.001, 1000 permutations) (Table 4).

### Putatively adaptive allele frequency changes are small

**Fig. 5:**
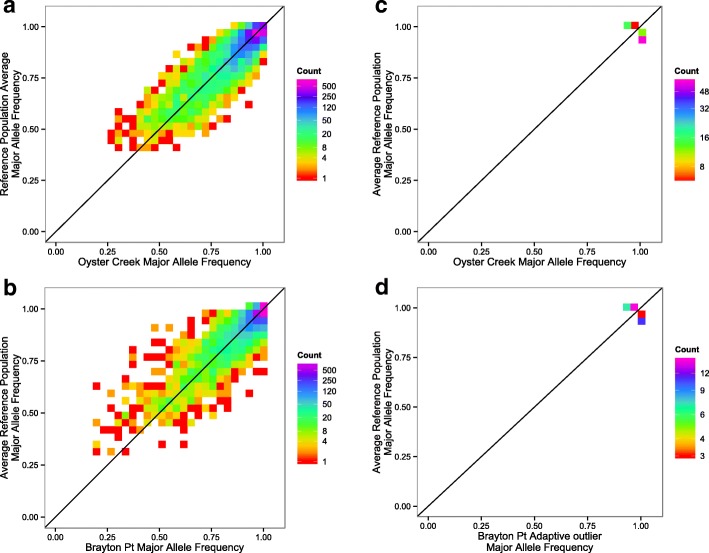
Allele Frequency Change Histograms. Allele frequency of the global major allele (of all 6 populations) in TE populations vs. the mean of both reference populations for all 5449 SNPs for the Oyster Creek triad (**a**) and the Brayton Point triad (**b**). The same for only the 94 candidate loci in Oyster Creek (**c**) and the 36 candidate loci in Brayton Point (**d**)

We examined the distribution of all allele frequency differences between reference and TE populations (Fig. 5a and b). Major allele frequencies are similar for both TE and reference populations (Fig. 5). There were no fixed differences between populations. In fact, all alleles at fixation in any one population were the major allele overall, both within and among the two triads. For each TE population and its two references, change in allele frequencies for all SNPs have a maximum of 33% change for Oyster Creek and a maximum of 45% for Brayton Point. At the majority of loci (90%), allele frequency differences are less than 10% between the Oyster Creek TE population and the mean of both Oyster Creek reference populations. Similarly, 92% of loci have less than 10% allele frequency differences for the same comparison in the Brayton Point triad.

For the candidate loci, the allele frequencies changes are also small. Among candidate loci, the maximum allele frequency change is 8% between the TE population and both reference populations for both the Oyster Creek and the Brayton Point triads (Fig. 5c and d). Putatively adaptive alleles (those favored in the effluent population) are rarely private alleles globally because most are the major allele overall, or the adaptive minor allele in the TE population is observed as the minor allele in the other triad.

### No extended blocks of elevated F_ST_ or LD around candidates

**Fig. 6:**
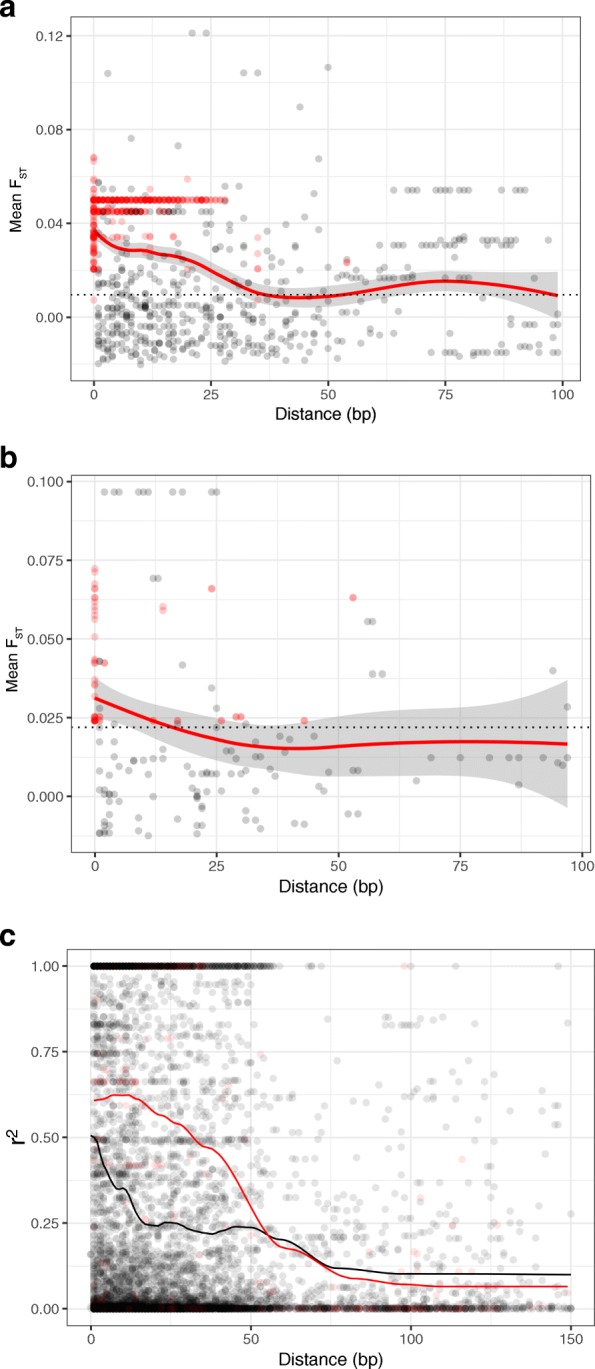
Decay of F_ST_ and LD near candidate SNPs. **a** and **b** Mean reference vs. effluent F_ST_ values at SNPs physically near candidate SNPs for the Oyster Creek triad (**a**) and Brayton Point triad (**b**). Distance is presented in base pairs from candidate SNP, smoothing line (red) is the Loess-smoothed mean F_ST_ value with 95% confidence intervals, dashed line is the mean genome-wide F_ST_ value estimate (dashed black line) for both reference vs effluent comparisons within the triad, red SNPs are other candidate loci. **c** Decay of linkage disequilibrium (r^2^): R^2^ among single SNP pairs, with loess smoothing line for SNP pairs that contain a candidate (red) and those that do not (black)

We examined the decay of mean pairwise F_ST_ values of effluent vs. reference comparisons for SNPs on the same genomic scaffold as candidate loci (Fig. 6, Additional file 7: Figure S5). The genomic regions with elevated F_ST_ values surrounding candidate loci are exceptionally small: < 30 bp. That is, the loess smoothed mean F_ST_ values of SNPs near candidates become indistinguishable from genome-wide mean F_ST_ after ~ 30 bp, for the Oyster Creek triad (Fig. 6a). For Brayton Point, elevated F_ST_ value regions near candidates are smaller; they are indistinguishable from genome-wide mean F_ST_ after ~ 18 bp (Fig. 6b). The extent of differentiation near candidate SNPs is also more similar to the genome-wide average for Brayton Point; these populations are more differentiated from each other on average across the genome (Table 1), but there is not a correspondingly large increase in the F_ST_ values reached by outliers.

Decay of linkage disequilibrium (R^2^) among all SNPs occurred on a similar spatial scale to the decay in F_ST_values for both triads, with smooth mean R^2^ falling to 50% of its maximum within ~ 30 bp (Fig. 6c, Additional file 8: Figure S4). The distance over which LD between pairs of SNPs decays to background levels is similar regardless of whether they contained a candidate, although LD was stronger in the vicinity of candidates than non-candidates at shorter distances (less than ~ 50 bp). Furthermore, no SNP was in significant LD (BY-FDR < 0.1) with a candidate at a distance greater than 1 kb.

While data at longer distances are sparse compared to data within about twice the distance of the sequence tags used to generate our dataset (128 bp), we also conducted a similar analysis at larger genomic scales (Additional file 7: Figure S5). We found no evidence of islands of elevated F_ST_ values at scales beyond 50 bp. Beyond 50 bp and up to 1 megabase from candidate loci, the 95% confidence interval of the smoothed mean F_ST_ value remains below the genome-wide mean level of differentiation between each of the two pairs of effluent vs. reference comparisons within the triad.

### Annotation term enrichment

We conducted a enrichment analysis of annotation terms to assess whether loci identified as candidates may have roles in biological pathways or processes that are canonically involved in thermal adaptation (Additional file 9: Table S2). Approximately half of sequence tags with adaptive SNPs mapped with genes in the DAVID database: 42 of 94 candidates for Oyster Creek map to the database and 21 of 36 for Brayton Point. One cluster of similar functional annotation terms was significantly enriched (EASE score > 1.3) in each triad: GTPase regulator activity in Brayton Point and protein complex assembly in Oyster Creek. However, several gene annotation clusters are enriched at lower EASE scores (0.5–1.2) and are salient because of their association with thermal adaptation. Functional annotation clusters for synaptic transmission and neuronal morphogenesis are enriched in both triads.

### Critical thermal maximum

To assess whether exposure to thermal effluents has led to an increase in thermal tolerance, we measured critical thermal maxima (CT_max_) in *F. heteroclitus* collected from Oyster Creek and its northern reference population after acclimation to laboratory conditions. Oyster Creek individuals demonstrated a significant, but minor increase in thermal tolerance relative to the northern reference population (ANOVA, *p* = 0.0483, *N* = 98). The critical thermal maximum for Oyster Creek was 39.7 ± 0.07 °C (mean ± s.e.) while the critical thermal maximum for the northern reference was 39.5 ± 0.07. °C. These differences correspond to a P_ST_ (phenotypic divergence in a trait across populations) value of 0.67, which exceeds F_ST_ among these populations (F_ST_ = 0.01), suggesting that selection may drive this difference in thermal tolerance. However, divergence in genetic architecture and narrow-sense heritability for this trait may also drive the observed divergence. To examine the robustness of the P_ST_-F_ST_ comparison to these effects, we bootstrapped sampled the data according to [58]. This analysis suggests divergence in the trait values exceeds expected divergence under genetic drift given the extent of genetic differentiation among these populations (Additional file 10: Figure S7).

## Discussion

Much of our theoretical understanding of adaptive evolution assumes a new mutation that rises to fixation quickly after the onset of selection [1]. Yet, much of adaptive evolution may involve neither new alleles, nor fixation of a single allele [20]. This latter scenario is particularly likely in natural populations with large effective population sizes, when selection is recent and the selection target is highly polygenic [10, 59]. Rapid evolution of whole organism performance is particularly salient to concerns regarding populations’ abilities to cope with rapidly changing environmental conditions. To characterize the genomic signature of rapid adaptation, we analyze changes in allele frequencies associated with recent thermal adaptation in two sets (triads) of populations. First, we examine demographic relationships among our sampled populations to understand the impacts of neutral processes on allele frequency differences within a triad. Then we establish that allele frequencies at a subset of the most differentiated loci within a triad do not follow a pattern parsimoniously explained by demographic processes, suggesting that selection unique to the thermal effluent environment may drive the differentiation at some loci. We then identify candidate loci using our triad experimental design in conjunction with an outlier analysis and further assessed that these candidate loci may be subject to selection through enrichment analysis of gene annotation terms and an examination of nucleotide diversity. Finally, we describe the signature of this putative selection on linked genetic variation near the candidate loci.

### Evidence of recent selection in response to thermal effluents

#### Population genetic structure

Using STRUCTURE on both the full SNP dataset and a set of putatively neutral SNPs, we find no evidence of strong population genetic structure in either triad, mean F_ST_ within a triad is very low and there is no significant pattern of isolation-by-distance at this genome-wide view. Yet several lines of evidence reduce our confidence in this conclusion: the Mantel tests used to test for IBD within a triad have limited power owing to the small size of the matrices [60], STRUCTURE is relatively poor at revealing structure at very low levels of differentiation [61], after removing correlated SNPs, STRUCTURE resolves each of teh Brayton Point triad populations into their own clusters, the AMOVA reveals significant among population variation within triads and the RDA demonstrates that spatial autocorrelation contributes significantly to a small portion of genetic variation within a triad. Taken together, these results suggest that for most loci, historic gene flow among our sampling locales should limit the extent of allele frequencies variation among populations within a triad. However, at the most differentiated loci, we expect a different pattern. In our sampling design, each effluent-affected population is flanked by two reference populations. For genomic regions subjected to only neutral or demographic forces, the expectation is that the two reference populations will demonstrate greater allele frequency differences than between a reference population and the intermediately located TE population. Patterns of genetic variation where the two more distantly located reference populations are more similar to each other than either is to the TE population are difficult to account for under neutral scenarios and may be due to selection unique to the TE population [51]. While we attribute these non-neutral patterns of divergence between TE and both reference populations to the temperature changes near the thermal effluents, other environmental or ecological factors could also be important.

We evaluate the neutral assumption of population genetic structure using three approaches. First, we utilize a model-based Bayesian approach (STRUCTURE) applied to the loci identified as outliers in any one of the pairwise comparisons within a triad. Thus we use the most differentiated loci to reveal subtle population genetic structure at loci whose differentiation is potentially influenced by selection [62]. Such an approach is frequently used to describe population structure when differentiation at most markers is low, but selection maintains differentiation at a subset a loci [63–65], despite violation of some model assumptions. To complement these results, we conduct two multivariate analyses on the full SNP datatset: a discrimination analysis (DAPC) [56] that maximizes the weighting of allele frequencies among principal components of genetic variation to describe differences among populations and redundancy analysis (RDA) to characterize differences among populations along orthogonal principal components of genetic variation and to examine the extent to which spatial autocorrelation and/or the presence of effluents significantly contributes to patterns of genetic variation among populations.

For the Oyster Creek triad there are two genetic clusters among outliers inferred by STRUCTURE. Individuals from the two reference populations primarily derive their ancestry at the most differentiated loci from one cluster, while the TE population is dominated by a second cluster. This pattern is consistent across the range of ancestral genetic clusters that we model and leads us to reject the neutral hypothesis for genetic variation among the most differentiated loci. The DAPC analyses support this finding. The second axis in DAPC using the full SNP dataset indicates a non-neutral pattern where the TE population is distinct from both reference populations with little distinction between the two reference populations. This divergence in the TE population in the second axis is different from the primary axis of genetic variation that follows a neutral pattern, where the position along the genetic axis of a population correlates with its geographic distribution. These patterns among all SNPS are expected if selection is occurring at the effluent site; we expect a mosaic of historic evolutionary forces to drive the differences observed among SNPs randomly sampled along the genome, with older, neutral forces shaping the majority of variation, but recent selection unique to the effluent site driving differentiation at some loci in the standing genetic variation.

In the Brayton Point triad, populations are more strongly differentiated (F_ST_ values ~ 0.03) than the Oyster Creek triad (F_ST_ values ~ 0.01), and STRUCTURE analysis using the most differentiated loci suggests that each population is unique. When we examine all loci for the Brayton Point triad using DAPC, however, we find a similar pattern as in the Oyster Creek triad. The major axis of genetic variation separates each of the populations in a pattern consistent with its geographic position, but the two reference populations are more similar to each other along the second major axis of genetic variation than either is to the TE population. In both the Oyster Creek triad and Brayton Point triad, the second largest component of genetic variation among the populations occurs in a pattern that is not consistent with neutral evolution. Therefore, we interpret the DAPC results as evidence of selection in both TE populations but caution that the STRUCTURE results do not corroborate this interpretation for Brayton Point where population differentiation is stronger.

To further examine the possibility that genetic variation within a triad is explained by both demography and the presence of effluents, we conducted three sets of partial RDAs: a set of global RDAs using both triads and a set of RDAs within each triad. Permutation of the genetic data in the RDA using data from both triads demonstrates that both spatial autocorrelation and effluents significantly contribute to genetic variation at this spatial scale. This result suggests that both effluent driven selection and isolation by distance influence allele frequencies to some extent. Yet, the model at the global spatial scale appears to underfit the genetic data. The portion of genetic data constrained by the model is much less than the variation among populations revealed by the AMOVA and average genome-wide divergence estimated by F_ST_. One possible explanation of this underfitting is non-linear relationships between the dbMEMs and historical restrictions of gene flow across the six populations. However, we do not have sufficient spatial sampling density to further examine this hypothesis and instead restrict our discussion to results from the two RDAs within triads.

The results for the RDAs within a triad are similar for both Brayton Point and Oyster Creek. In both triads, effluents and spatial autocorrelation each significantly explain variation in the genetic data once controlling for the other using pRDA, corroborating the findings obtained through STRUCTURE on highly differentiated loci and DAPC on the full SNP dataset. After a pattern of spatial autocorrelation or IBD, effluents explain a portion of the genetic variation among populations. It is important to underscore here that RDA is correlative. RDA finds linear combinations of the explanatory variables that are redundant with, i.e. linearly correlate with, linear combinations of the response variables. Given that the spatial autocorrelation variables should place the effluent triad intermediate to the two reference sites, while the effluent variable separates the effluent site from the two reference sites, the RDA within a triad recapitulates the triad approach used with DAPC and STRUCTURE to identify putatively non-neutral patterns within a triad. RDA reveals whether patterns of genetic variation within a triad conform to the expectation if selection unique to the effluent sites is driving allele frequency variation once we account for demographic patterns. While RDA does not establish a causal link between this potentially adaptive variation and selection, it is a powerful approach because unlike DAPC or STRUCTURE it is (i) capable of determining the portion of overall genetic differentiation potentially explained by demography and selection at the effluent site through variance partitioning and (ii) provides a statistically robust framework to infer whether these patterns might arise by chance alone.

#### Oyster Creek critical thermal maxima

Although the three populations within the Oyster Creek triad demonstrate little genetic divergence, critical thermal maximum (CT_max_) of individuals from the effluent impacted habitat was significantly higher than that of individuals from the northern reference population. Assuming a correlation between CTmax performance in the laboratory and thermal tolerance in the wild, these data suggest that Oyster Creek individuals are phenotypically adapted to the effluent impacted environment. *F. heteroclitus* populations separated by 1000 s of kilometers demonstrate compensatory variation in CT_max_ (New Hampshire and Georgia populations, ~ 0.6 °C change in thermal tolerance) when individuals are acclimated to similar temperatures as those used in our analysis [66]. Thus, these results are best interpreted with caution because our nearby southern *F. heteroclitus* population is expected to be marginally more thermally tolerant due to its location ~ 30 km kilometers south along the coast from the reference population. Additionally, our analysis cannot rule out irreversible thermal acclimation or developmental plasticity. Nor did we conduct a similar analysis within the Brayton Point triad, where population genomic evidence of possible selection at the effluent site is weaker. However, it seems unlikely that clinal adaptation would lead to the observed variation between the Oyster Creek TE and the northern reference population, which is only separated by ~ 30 Km, when the magnitude of this difference is approximately one third of the difference among populations separated by thousands of kilometers. Instead, we suggest that the Oyster Creek population difference is more likely due to adaptive genetic or non-reversible plastic responses to the thermal effluent. P_ST_-F_ST_ comparisons support this conclusion. The degree of phenotypic divergence among populations exceeds that expected that might arise solely due to drift given the extent of genome-wide divergence among the populations across a wide parameter space of potentially varying genetic architecture [58].

### Signature of recent selection in response to thermal effluents

#### Candidate loci identification

Separating true signals of directional selection from the extreme tails of neutral variation is a persistent challenge associated with genomic outlier scans [67, 68]. Outlier scans suffer from both Type I and II error to varying extents depending on the demographic history of the populations in question. In particular, departures from the island model of migration such as spatial autocorrelation of allele frequencies due to isolation by distance (IBD) or expansion from refugia can lead to high false positive rates [68]. Within a triad, we do not observe significant IBD using a Mantel test and the degree of differentiation is small, however, the RDA demonstrates that spatial autocorrelation may contribute to differentiation. To more conservatively identify potential adaptive divergence in TE populations, we again utilize the triad sampling design (Fig. 1). In our analysis we define candidate loci as those that are significant outliers in both TE population versus reference population comparisons but are not outliers between the reference populations. Our definition of candidate loci then combines the typical F_ST_-based outlier approach with additional requirements (Fig. 3).

To assess whether candidate loci have annotations that provide insights into the genes responsible for thermal adaptation, we conducted an enrichment analysis. Two observations bolster the conclusion that variation at the candidate loci may lead to increased thermal tolerance and strengthen the evidence that the candidates contain loci in linkage disequilibrium with true targets of selection. First, candidate loci are enriched for several annotation terms that are canonically associated with thermal adaptation. For example, immunoglobulin, apoptosis, and plasma membrane structure genes are consistently observed as thermal adaptation targets in fish [48, 69] and are loci with related annotation terms are enriched among the candidate loci. Second, there is some evidence of adaptive convergence in gene annotation terms that are enriched in both TE populations. Candidate loci from both triads demonstrate non-significant enrichment for annotation terms associated with synaptic transmission and neuronal morphogenesis. Interestingly, these functional annotation clusters shared among triads have typically not been implicated in fish thermal adaptation and highlight the advantage of taking a functionally agnostic approach to investigating thermal adaptation.

Next, we consider variation at and around these candidate loci to examine the genomic signature of recent selection due to thermal effluents. In particular, we address three questions (i) are adaptive shifts in allele frequency between reference and effluent-affected populations large or small, (ii) are adaptive alleles common or rare in the standing genetic variation, and (iii) are candidate loci embedded in extended genomic regions of elevated differentiation and linkage disequilibrium, or are these islands of differentiation near candidate loci limited to the scale of ancestral linkage disequilibrium?

#### Examining the signature of putative selection

In the classical paradigm, adaptation proceeds through selective sweeps that drive an advantageous allele from low to very high frequency. However, evidence from quantitative genetics [22, 70, 71], population genetics [72–75] and association genetics [19, 76, 77] suggests that subtle allele frequency shifts across many variants (polygenic adaptation) also play an important role in evolution. On its face, the observation that candidate loci demonstrate only small allele frequency shifts between reference and TE populations suggest that polygenic selection has played a role in this case of recent selection in large natural populations. However, two alternative sweep scenarios may account for the subtle allele frequency differences at candidate loci between effluent and reference populations. First, gene flow from populations under selection may have driven alleles to high frequency in nearby populations where they are in migration-drift equilibrium. In this scenario, a rare allele in the TE population was driven to high frequency due to natural selection since the onset of selection at the effluent site, and introgression of this previously rare allele into the reference populations has altered the reference population allele frequencies. Given the low degree of differentiation, and thus high gene flow within a triad, this scenario may explain the subtle allele frequency shifts; introgression of adaptive alleles from the effluent population should be rapid and homogenize allele frequencies. However, this scenario does not readily account for alleles that are fixed in the reference populations but present at lower frequencies in the TE populations (i.e.*,* directional selection in favor of the triad minor allele). Furthermore, in the minority of cases where the minor allele is putatively advantageous in the TE environment (minor allele has higher frequency in the TE population among 33% of candidate loci), it is rarely a private allele among all six populations in both triads, and is therefore likely available in the standing genetic variation either below our minor allele frequency filtering cutoff, or low enough in frequency that it is not included in our sample by chance, given the only moderate genetic distance between populations in different triads and large population sizes. We conclude that at the majority (98%) of candidate loci, the adaptive allele is likely present in the standing genetic variation, either because it is the major allele overall or it is present at an appreciable frequency in populations with appreciable gene flow (Φ_CT_ = 0.096).

In the second scenario, candidate loci may be in linkage disequilibrium with, but far from, those loci actually driving selection (i.e. the candidate loci are on “soft shoulders”) [78]. Both recombination and mutation during and after a selective sweep can violate the simplifying assumption of a monotonic increase in test statistics as the distance to the locus under selection decreases. This stochasticity can lead to peaks in test statistics that are not centered over the locus under selection, suggesting that our candidate loci may be peaks of F_ST_ embedded in the shoulders surrounding a hard sweep. We expected that if variation at candidate loci is due to a selective sweep, candidates would lie in large regions with elevated F_ST_ values due to the fixation of rare haplotypes bearing the adaptive allele, and that LD would be high along these regions [79]. Our data does not fit this pattern: F_ST_ and LD decay fully to genome wide averages within very short scales. The region surrounding a candidate where the F_ST_ value exceeds the genome-wide average extends less than 50 bp, a similar scale to the decay of LD (R^2^) for the full SNP dataset (Additional file 4: Figure S3) and we find no extended blocks of high linkage disequilibrium surrounding candidate SNPs. Nor do we find any SNPs in significant linkage disequilibrium with candidates at distances greater than 1 kb.

We do not have an example of an elevated F_ST_ value region due to a hard sweep in *F. heteroclitus* to compare our results to, but observations in other species suggest that the LD distance we find is orders of magnitude shorter than any predicted unit of adaptive hitchhiking due a recent selective sweep. An empirical estimate of the average unit of adaptive hitchhiking in humans is 20 kb, where F_ST_ values are highly significantly correlated [80]. Simulation studies suggest these signals can extend up to 200 kb [78]. Furthermore, genome scans that rely on a moving average of F_ST_ values commonly utilize windows of 100 kb or more (e.g.*,* [81]). Taken together, the rapid decay of F_ST_ and LD suggest that selection has likely acted on adaptive alleles at the candidate locus that coalesce well before the onset of selection and multiple haplotypes bearing the adaptive allele are under selection. This pattern has been recognized as a consequence of rapid polygenic evolution in other species with large population sizes [75, 82].

### Polygenic selection

Under polygenic adaptation, selection drives many subtle shifts in allele frequency until a new phenotypic optimum is reached. Our data implicate a model of the early stage of adaptive evolution where selection acts on previously segregating mutations to produce small changes in allele frequency at many loci: putatively adaptive alleles are present at appreciable frequency in nearby populations, adaptive variation in allele frequency is small, and regions of elevated F_ST_ values surrounding these alleles decay at a distance on a similar scale to genome-wide average patterns of linkage disequilibrium suggesting they are derived from the standing genetic variation borne on several haplotypes. Therefore, if our candidate loci contain SNPs in LD with the true targets of selection, recent thermal adaptation in these populations is consistent with polygenic adaptation in that adaptive alleles are borne on multiple haplotypes that coalesce before the onset of selection (as is the case under a soft sweep), but the adaptive alleles themselves are not swept to high frequency.

While the parameter space selection on the standing genetic variation producing soft sweeps become feasible may be narrow [47], there are a number of reasons to expect that adaptation to the Oyster Creek and Brayton Point thermal effluents should produce the pattern of polygenic adaptation from standing genetic variation that we observe. First, selection due to thermal effluents has occurred for approximately 50 generations, and adaptive polymorphisms are more likely to be derived from the standing genetic variation when selection is quite recent because there has been insufficient time for new mutations to arise [83, 11]. Simply put, there hasn’t been enough time for a sweep of any kind to occur. Selection on standing variants is also more likely to occur where the effective population size is large [10]. Estimates of *Fundulus heteroclitus* effective population sizes are large, ranging from 2X10^4^ [84] to 3X10^5^ [85]. Effective population size in TE populations should be similar to these estimates because we do not find substantially reduced genetic diversity relative to the overall diversity. Finally, adaptation from the standing genetic variation is only likely where the adaptive allele is nearly neutral and present at an appreciable frequency before the onset of selection [86]. Most of our SNPs identified as adaptive in this analysis are the major allele overall in all six populations, and those that are not occur at moderate frequency in moderately distant populations. Finally, unlike other cases of rapid evolution that are predicted to produce local hard sweeps because of the limited mutational target size [44, 47], the mutational target size of thermal adaptation is likely quite large [48, 49]. Consequently, the effective value of θ = 2N_e_μ_a_ (where μ_a_ is adaptive mutation rate) and therefore the probability of adaptation from the standing genetic variation, is increased in the case of thermal adaptation [10, 59].

It is important to also emphasize that our data do not preclude the possibility of sweeps, hard or soft, as the final outcome of selection in these effluent populations, nor do they preclude a role for genomic islands of differentiation. Absence of evidence is not evidence of absence. We surveyed the genetic variation at a small subset of the total polymorphisms present and cannot refute other patterns of variation at loci that our dataset does not query. In fact, moderate genome size in *F. heteroclitus* (~ 1.26 Mb) [87], coupled with observed LD extending only hundreds of base pairs, and the small number of markers that exceed our filtering parameters together suggest that the proportion of the genome effectively tagged by our dataset is potentially as small as 1–5% [88]. Also, it is likely we do not observe a sweep because selection in these populations is ongoing and there has been insufficient time for the fixation of adaptive alleles [89–91].

There is a fundamental disconnect between the time scale of common models of molecular evolution and rates of phenotypic adaptation observed in natural populations [92] and in experimental evolution [93]. The significance of rapid adaptation to anthropogenic stressors and species introductions demands an increased understanding of the population genetic basis of evolution in these cases of rapid evolution. Polygenic selection has been suggested as an explanation of this discrepancy because it provides a mechanism of rapid adaptation without the fixation of adaptive alleles [20]. Specifically, as the genetic architecture of a trait becomes increasingly polygenic, with each locus bearing a smaller phenotypic effect size, the probability of fixation decreases [21] and rates of adaptive phenotypic evolution can become rapid [23]. Thus, subtle shifts in allele frequency at many loci underlying trait variation may lead to adaptation at the population level provided effect size for each locus is small. This effect size distribution may be likely for complex fitness traits [18, 94].

## Conclusion

We combined a population sampling regime that allows us to identify potentially adaptive variation with an analysis of population genetic structure at varying levels of differentiation and F_ST_-based outlier scans. We used this approach to assess the hypothesis that populations exposed to thermal effluents near coastal power stations experience recent selection and then examined the genomic signature of this putative recent selection to investigate the evolutionary history of adaptive alleles. We conclude that fish living near thermal effluents have rapidly evolved from the standing genetic variation through small allele frequency changes at many loci in a pattern consistent with a polygenic model of evolution. Overall, we suggest that evolution through these mechanisms may be a common feature early in the adaptive process in large, outbred, natural populations exposed to environmental changes with broad physiological impacts, but caution that our low resolution genomic dataset may not tell the full story in these populations and that polygenic effects are likely to be only transient in nature.

## Methods

### Populations

*F. heteroclitus* were collected from six sites. The six locations form two triads, where a triad contains a single population subjected to thermal effluents (TE) as well as northern and southern reference populations (Fig. 1). For the Oyster Creek triad, the TE population was sampled along Oyster Creek, in Forked River, NJ (39°48′31.40″N, 74°11′3.72″W), the northern reference population was sampled at Mantoloking, NJ (40°3′0.02″N, 74°4′4.92″W), and the southern reference population was sampled at the Rutgers University marine field station in Tuckerton, NJ (39°30′31.60″N, 74°19′28.11″W). For the Brayton Point triad, the TE population was sampled at a marsh ~ 1 km from the effluent canal (41°42′44.99″N, 71°11′9.74″W), the northern reference population was sampled at Horseneck Beach, MA (41°30′16.16“N, 71° 1’32.03”W), and the southern reference was sampled at Matunuck, RI (41°22′56.45″N, 71°31′32.04″W). Fish were captured using wire mesh minnow traps. Fin clips were taken for GBS (genotyping by sequencing) library preparation; other fish from Oyster Creek and Mantoloking, NJ were transported live to the laboratory in aerated seawater for later critical thermal maximum analyses.

Fieldwork was completed within publically available lands and no permission was required for access. *F. heteroclitus* does not have endangered or protected status, and do not require collecting permits for non-commercial purposes in the sampling locations. All fish were captured in minnow traps and removed within 1 h. IACUC approved procedures were used for non-surgical tissue sampling.

### GBS library preparation and population genetic analysis

Fin clips from the terminal margin of the caudal fin approximately 5 mm^2^ in size were taken from individuals in the field using scissors and stored in 270 ul of Chaos buffer (4.5 M guanadinium thiocynate, 2% N-lauroylsarcosine, 50 mM EDTA, 25 mM Tris-HCl pH 7.5, 0.2% antifoam, 0.1 M β-mercaptoethanol); these samples were stored at 4 °C prior to processing. Genomic DNA was isolated from fin clips using an Epoch Life Sciences silica column [95]. DNA quality was assessed via gel electrophoresis and concentrations were quantified in triplicate using Biotium AccuBlueTM Broad Range dsDNA Quantitative Solution according to manufacturer’s instructions. 100 ng of DNA from each sample was dried down in 96-well plates. Samples were then hydrated overnight with 5 ul of water before restriction enzyme digestion and further processing.

Genomic DNA (gDNA) was isolated from 296 individuals. These gDNA samples were individually barcoded and used to create a reduced representation library for genotyping by sequencing (GBS) [96]. The library was created in duplicate with barcode assignment of individuals randomized across both replicate libraries. Each replicate library was sequenced on 1 Illumina Hi-Seq2500 lane. The combined raw dataset consisted of 274,102,532 single-end, 75 bp reads. The reference genome-based GBS analysis pipeline, TASSEL [97] was used to call SNPs using the *Fundulus heteroclitus* genome [46]; SNPs were identified using the “Discovery Build.” In brief, TASSEL collapses identical, individually barcoded, short sequence reads, aligns these reads to a reference genome and calls genotypes on the basis of allelic redundancy using up to 127 reads per unique sequence tag per individual. Reads beyond this depth are ignored. Heterozygotes are called using a binomial-likelihood approach [98]. A log of console input for the pipeline is available upon request. We largely used default settings throughout the pipeline with the following exceptions: a minimum of 5 counts were required for retention of unique tags during the merge multiple tag count fork, and tag alignment to the reference genome was accomplished with bowtie2 using the very-sensitive-local setting.

We found 1,451,801 unique sequence tags that contained both the barcode and *AseI* cut site. Bowtie aligned 1,142,340 (78.7%) of these tags to unique loci in the *F. heteroclitus* genome [46]; 159,591 (11.0%) sequence tags aligned to multiple loci, and 149,870 (10.3%) had no alignment. The latter two tag sets were excluded from further analysis. Heterozygotes were called using a binomial likelihood ratio based approach of quantitative genotype calling, as implemented in the TASSEL-GBS discovery pipeline [98]. Among the 1.1 million tags that singly aligned to the *F. heteroclitus* genome, we identified 314,746 SNPs.

We filtered the 314,746 SNPs identified by the TASSEL discovery pipeline among all 296 individuals in the library. We removed fifty-seven individuals missing more than 12.5% of SNPs. To remove polymorphisms that may have arisen from sequencing and amplification errors or alignment across paralogs (versus polymorphisms between alleles), we then filtered the SNP dataset by coverage, minor allele frequency and whether observed heterozygosity (H_o_) was significantly greater than the expected heterozygosity (H_e_) [99, 100]. Retaining SNPs that were called in at least 85% of the remaining 239 individuals, resulted in 5907 SNPs. Of the 5907 SNPs in 239 individuals, 110 with minor allele frequencies less than 1% were removed. Hardy-Weinberg equilibrium was calculated for individual loci using Arlequin v3.5.1.2 [101] using 1000,000 steps in the Markov chain with 100,000 dememorization steps. Then, 348 SNPs with H_o_ > H_e_ that exceeded Hardy-Weinberg equilibrium at *p* < 0.01 were removed. Thus, the fully filtered SNP dataset consisted of 5449 SNPs among 239 individuals. Mean read depth per SNP per individual was 26.29 ± 0.43 for the full SNP dataset (Additional file 1: Figure S1 and Additional file 11: Figure S6). Most (64.3%) SNPs in the final full SNP dataset have at least 10 reads in all individuals. The TASSEL-GBS pipeline caps the number of reads used to make a call at 127 for each allele. Therefore, the range of read depth per individual per SNP in the dataset was 0–254 (2*127), and the high frequency of 127 and 254 counts per SNP per individual in the read depth frequency distribution results from highly sequenced individual–by-SNP combinations.

Outlier scans were conducted with FDIST2 [102] as implemented in LOSITAN [53]. For all pairwise population comparisons we used the same settings. We culled loci that are potential outliers to more narrowly estimate initial mean F_ST_ values (neutral mean F_ST_ option) and used the bisection approximation algorithm to estimate mean F_ST_ values (force mean F_ST_ option). After these steps, we conducted 50 k simulations. To control for multiple comparisons, we adjusted empirical *p*-values provided by LOSITAN with a modified FDR of 5% [54, 55]. These results were also used to construct putatively neutral panels of SNPs in each triad for population genetic inference. This dataset includes SNPs that are not outliers due to either putative directional or balancing selection (i.e. 0.05 < p-simulated < 0.95) in any of the three pairwise comparisons within a triad.

Population genetic parameters were calculated in a variety of statistical packages. Isolation by distance was tested using a Mantel test (9999 simulations) in the ade4 R package [103]. Arlequin v3.5.1.2 was used to calculate the proportion of inter-population pairwise differences, intra-population estimates of nucleotide diversity (π), and AMOVA (99,999 permutations). Linkage disequilibrium (r^2^) and sliding window nucleotide diversity was calculated in the tassel GUI. Smoothing conditional mean r^2^ and F_ST_ along the genome was accomplished using LOESS. The span for each fit was chosen using the bias-corrected Akaike information criterion [104].

Population genetic structure inference was made using STRUCTURE [105] and DAPC [56]. STRUCTURE analyses were performed on five subsets of the data: (a) the complete SNP dataset within each triad (effluent population + two reference populations), (b) a LD thinned dataset within each triad consisting of 3992 SNPs created by randomly removing one SNP from any pair with r^2^ > 0.5 (c) a “neutral” SNP dataset with both potential directional and balancing selection outlier loci (*p*–value: 0.05) excluded, (d) a SNP dataset consisting of all pairwise directional selection outliers within a triad, i.e. the most differentiated loci among the population within a triad and (e) a minor allele frequency matched neutral dataset. For (e), we bootstrap sampled the “neutral” dataset for each triad, so that the allele frequency distribution matched that of the dataset composed of the most differentiated loci. For all analyses, we used a burn in of 10 k steps and MCMC of 20 k steps with at least 7 replicates for each *k* value and varied *k* from 1 to 6. In all replicates, burn in was sufficient for convergence in the STRUCTURE parameters: α, F, D and likelihood. We specified an admixture model with correlated allele frequency to improve clustering among closely related populations within a triad [105]. Replicate individual *k* runs were merged and the ΔK method for identifying the optimal number of clusters [106] was calculated using CLUMPAK [107]. For Oyster Creek, likelihood scores (Pr(X|K)) beyond *K* = 2 increase at a decreasing rate (Additional file 4: Figure S3a), and the Δ*K* method identifies *K* = 2 as the optimal number of population clusters (Additional file 4: Figure S3c). For Brayton Pt, although the Δ*K* method identifies *K* = 4 as the optimal number of population clusters, likelihood scores (Pr(X|K)) plateau at *K* = 3 (Additional file 4: Figure S3b and d), and results at *K* higher than 3 are qualitatively similar. The first genetic split (*K* = 2) separated the southern reference population (Matunuck, RI) from both the TE and the northern reference populations (Horseneck Beach, MA).

For DAPC, we used the full SNP dataset. For the Brayton Point triad, we identified 2 as the optimal number of principal components (PCs) (Additional file 4: Figure S3 h), although using up to 40 PCs produced qualitatively similar results. DAPC perfectly matched inferred genetic clusters with the three populations using these 2 PCs. Furthermore, the Bayesian information criterion (BIC) of K-means clustering from *K* = 1–12 demonstrated an inflection point at *K* = 3 (Additional file 4: Figure S3f). These results remained consistent when up to 40 PCs were used.

For the Oyster Creek triad, the *a-*score was low from 1 to 90 retained PCs; i.e.*,* individuals from single populations did not reliably fall into single genetic clusters (Additional file 4: Figure S3 g). Similarly, the BIC for successive *K*-means clustering suggested 1 as the appropriate number of genetic clusters to describe the data (Additional file 4: Figure S3e), despite the extent of population differences indicated by pairwise genome-wide F_ST_ value estimates (Table 1) and significant among population differences in the AMOVA (Table 2). As our goal was to use DAPC to describe the genetic structure among fish capture from the three sampling sites along orthogonal axes of genetic variation rather than to establish the extent of true population differences, we performed DAPC using population (sampling location) as the grouping factor rather than inferred genetic clusters, as is common. This analysis maximizes the differences among a priori assigned populations rather than among the putative genetic clusters contained in our dataset and therefore introduces the possibility of overfitting differences among a priori identified populations (sampling locations). Including more or less PCs in this DAPC did not change the relationship among populations from 5 to 90 PCs, but populations were more distinct with more PCs and therefore more genetic variation was incorporated into the DAPC. We retained the first 32 PCs.

We also examined the extent to which spatial and environmental (effluent) variables could be used to explain genetic variation among populations using redundancy analysis (RDA) and partial redundancy analysis (pRDA). RDA is a form of constrained ordination that can be used to describe linear relationships among components of multivariate response and multiple multivariate explanatory variables [57]. In pRDA, redundancy analysis is conducted on the residuals of the response variable after modeling the effect of one or more of the explanatory variables using RDA. In our RDA we used major principal components of genetic variation as the response variable, with the number of retained principal components determined by the Kaiser-Guttman criterion [108], and two explanatory variables: a spatial variable and the presence or absence of effluents. For the spatial variable we employed distance-based Moran’s eigenvector maps (dbMEMs). dbMEMs are capable of describing spatial variation at multiple scales, including spatial autocorrelation as well as local structures and are suitable as explanatory variables for constrained ordinations, such as RDA [109]. We retained all dbMEMs with positive values of Moran’s I as our spatial explanatory variables because we were interested in modeling only the effect of positive spatial autocorrelation on genetic variation using the spatial variable. Significance of the RDA is then tested using empirical *p*-values (permuting the response variables). We conducted the RDA using the R package *vegan* [110], using the *rda()* command and the anova.cca() command to test its significance. dbMEMs are calculated using a distance matrix of alongshore (5 m depth constrained) distances among sampling locations and the *dbmem()* command from the R package *adespatial* [111]. To calculate the proportion of variance explained by the constrained axes of the RDA, we used variance partitioning [112] implemented in the *varpart()* command of *vegan* in R. Because variation in PCA is equivalent to F_ST_ [113], we used our ordination results to calculate the proportion of among population variation explained by each of the models, using total constrained variation weighted by the portion of variation explained among the major principal components of genetic variation as the numerator and F_ST_ as the denominator [114]. To test the significance of each set explanatory variables in explaining genetic variation separately, we conducted pRDA, conditioning the genetic variation on each spatial and effluent variables, followed by a permutation analysis, again implemented in *vegan* using the *rda()* and *anova()* commands. We conducted three RDAs with pRDAs: a global RDA of both triads, and each triad separately.

Functional enrichment among candidate loci was assessed with DAVID v6.7 [115]. Candidate loci were annotated using the *F. heteroclitus* genome [46] where gene identifiers are based on expression and homology evidence. These gene identifiers were submitted to the Functional Annotation Clustering tool in DAVID v6.7 against a background of all *F. heteroclitus* gene annotations using the “high” classification stringency. DAVID’s Functional Annotation Clustering Tool groups similar functional annotations and collectively evaluates their enrichment. The statistical significance of annotation clusters is evaluated on the basis of DAVID’s EASE score: the mean value of the negative log-transformed, FDR corrected p-values of enrichment for all genes included in that cluster [115]. Clusters of enriched annotation terms with an EASE score greater than 1.3 are considered significant.

### Critical thermal maximum

Using separate *F. heteroclitus* collections from the Oyster Creek TE population (*n* = 51) and its northern reference population (Mantoloking, NJ) (*n* = 47), we measured upper thermal tolerance with the critical thermal methodology [116]. Fish were maintained in the laboratory in a recirculating seawater system containing less than 1 fish per gallon and fed daily in the afternoon. Salinity, ammonia and temperature were checked regularly. All protocols were approved by the institutional animal care and use committee (IACUC protocol 13–054). Fish were acclimated for 8 weeks to 28 °C and 15 ppt salinity using artificial seawater and a 14:10 h light:dark schedule to reduce the effect of reversible acclimatization to local field conditions.

The experimental chamber consisted of a 20 L aquarium within an insulated 40 L aquarium. Both chambers were filled with acclimation temperature water (28 °C), then 70 °C water was introduced from a header tank to the outer aquarium at a controlled rate to maintain heating at 0.28–0.30 °C/min in the inner tank throughout the experiment. The inner chamber was aerated to reduce thermal stratification and maintain oxygen saturation during trials. After acclimation, groups of six fish were introduced to the inner aquarium. Critical thermal maxima were determined based on continuous loss of equilibrium for 5 s. 99% of individuals survived the critical thermal maximum trial after 1 week. CTmax measurements were used to calculate the degree of phenotypic divergence among the populations (P_ST_) relative to overall genetic divergence (F_ST_). We bootstrapped these data to produce confidence intervals for P_ST_ across varying degrees of heritability and among population changes in genetic architecture using the R package *Pstat* [58].

## Additional files

Additional file 1: **Figure S1.** Depth of read per individual at each SNP in the 5.4 k SNP dataset. (A) Mean depth across individuals represented by red line. (B) Frequency histogram of reads per SNP per individual. (PDF 1857 kb)

Additional file 2: **Table S1.** AMOVA results for neutral dataset. (DOCX 38 kb)

Additional file 3: **Figure S8.** STRUCTURE results for after LD thinning the full dataset. STRUCTURE plots for LD thinned dataset (r^2^ < 0.5, 3992 SNPs) among (A) Brayton Point triad, best k = 3, and (B) Oyster Creek triad, best k = 1. (PDF 1589 kb)

Additional file 4: **Figure S3.** Supporting population genetic structure data. (PDF 820 kb)

Additional file 5: **Figure S2.** Outlier Scans. Lositan output with distributions of F_ST_ values and observed H for all within triad pairwise comparisons. (PDF 1080 kb)

Additional file 6: **Figure S9.** STRUCTURE results for after LD thinning the outlier dataset. STRUCTURE plots at best k for the most differentiated SNPs, LD thinned (r^2^ < 0.5) among (A) Brayton Point and (B) Oyster Creek triads. (PDF 1141 kb)

Additional file 7: **Figure S5.** Decay of F_ST_ away from candidate SNPs. Mean reference vs. effluent F_ST_ values at SNPs with 1mb and 100 kb from candidate SNPs for the Oyster Creek triad (a) and Brayton Point triad (b). Distance is presented in base pairs from candidate SNP, smoothing line is the Loess-smoothed mean F_ST_ value with 95% confidence intervals, dashed line is the mean genome-wide F_ST_ value estimate (dashed black line) for both reference vs effluent comparisons within the triad, dashed smoothing line (blue) is Loess-smoothed mean of a random permutation of distance vs. mean reference vs. effluent F_ST_ values, red SNPs are candidate loci. (PDF 3502 kb)

Additional file 8: **Figure S4.** Long Range LD Decay. R^2^ among single SNP pairs across the full dataset, with loess smoothing line for SNP pairs that contain a candidate (red) and those that do not (black). (PDF 649 kb)

Additional file 9: **Table S2.** Functional annotation clusters for Oyster Creek (top) and Brayton Point (below). For each cluster the EASE score, functional annotation terms, gene identifiers, *p*-value for individual annotation terms, and fold enrichment relative to the *F. heteroclitus* background are presented. (DOCX 26 kb)

Additional file 10: **Figure S7.** P_ST_-F_ST_ Comparison. P_ST_ and it’s 95% confidence interval for critical thermal maximum differences among *F. heteroclitus* from Oyster Creek and its northern reference population. P_ST_ is estimated for a range of *c/h*^*2*^*,* where c is proportion of the total variance that is presumed to be because of additive genetic effects across populations and h^2^ is narrow sense heritability. F_ST_ is plotted in green. P_ST_ exceeds F_ST_ for the majority of the parameter space, suggesting that the degree of phenotypic divergence exceeds that expected given the extent of genome-wide divergence alone. (PDF 194 kb)

Additional file 11: **Figure S6.** Filtering impacts on coverage summary. (A) Density plot of reads per SNP per individual for the filtered (green) and raw (purple) datasets. (B) Density plot of non-zero reads per unique tag per individual for filtered dataset (purple) and the raw dataset (green). (C) Density plot of reads per SNP per individual for the filtered (green) dataset and the raw dataset excluding with SNPs where > 75% of individuals demonstrate zero reads (blue). Summary: There are large differences in coverage between our filtered (5.4 k) and raw SNP (314 k) datasets. The median read depth per SNP per individual of the raw dataset (panel A, purple) is 0 (filtered data median = 16, green). The mean read depth per SNP per individual of the raw dataset is ~ 4.4 (filtered data mean = ~ 26.3). While we base our filtering on missingness across SNPs and individuals (missing genotype calls results from less than 5 reads per unique 64 bp sequence tag within an individual), the majority of SNPs we filter out have zero reads in the majority of individuals, but coverage similar to our filtered dataset in the remaining individuals (panel B and C). (PDF 746 kb)

## Abbreviations

AMOVA: Analysis of molecular variance
CTmax: Critical thermal maximum
DAPC: Discriminant analysis of principal components
IBD: Isolation-by-distance
LD: Linkage disequilibrium
pRDA: Partial redundancy analysis
P_ST_: Phenotypic divergence among populations
RDA: Redundancy analysis
SNP: Single nucleotide polymorphism
TE: Thermal effluent

## References

[1] Orr HA (2005). The genetic theory of adaptation: a brief history. Nat Rev Genet..

[2] Smith JM, Haigh J (1974). The hitch-hiking effect of a favourable gene. Genet Res.

[3] Nielsen R, Williamson S, Kim Y, Hubisz MJ, Clark AG, Bustamante C (2005). Genomic scans for selective sweeps using SNP data. Genome Res.

[4] Haasl RJ, Payseur BA (2016). Fifteen years of genomewide scans for selection: trends, lessons and unaddressed genetic sources of complication. Mol Ecol.

[5] Laurent S, Pfeifer SP, Settles ML, Hunter SS, Hardwick KM, Ormond L (2016). The population genomics of rapid adaptation: disentangling signatures of selection and demography in white sands lizards. Mol Ecol.

[6] Linnen CR, Kingsley EP, Jensen JD, Hoekstra HE (2009). On the origin and spread of an adaptive allele in deer mice. Science..

[7] Bock DG, Caseys C, Cousens RD, Hahn MA, Heredia SM, Hubner S (2015). What we still don't know about invasion genetics. Mol Ecol.

[8] Schloss CA, Nuñez TA, Lawler JJ (2012). Dispersal will limit ability of mammals to track climate change in the Western hemisphere. Proc Natl Acad Sci.

[9] Hendry AP. Eco-evolutionary dynamics. Princeton: Princeton University press; 2016.

[10] Pennings PS, Hermisson J (2006). Soft sweeps II--molecular population genetics of adaptation from recurrent mutation or migration. Mol Biol Evol.

[11] Hermisson J, Pennings PS (2005). Soft sweeps: molecular population genetics of adaptation from standing genetic variation. Genetics..

[12] Messer PW, Petrov DA (2013). Population genomics of rapid adaptation by soft selective sweeps. Trends Ecol Evol.

[13] Terekhanova NV, Logacheva MD, Penin AA, Neretina TV, Barmintseva AE, Bazykin GA (2014). Fast evolution from precast bricks: genomics of young freshwater populations of threespine stickleback Gasterosteus aculeatus. PLoS Genet.

[14] Garud NR, Petrov DA (2016). Elevated linkage disequilibrium and signatures of soft sweeps are common in Drosophila melanogaster. Genetics..

[15] Raquin AL, Brabant P, Rhoné B, Balfourier F, Leroy P, Goldringer I (2008). Soft selective sweep near a gene that increases plant height in wheat. Mol Ecol.

[16] Jones BL, Raga TO, Liebert A, Zmarz P, Bekele E, Danielsen ET (2013). Diversity of lactase persistence alleles in Ethiopia: signature of a soft selective sweep. Am J Hum Genet.

[17] Swallow JG, Hayes JP, Koteja P, Garland T Jr. Selection experiments and experimental evolution of performance and physiology. Exp Evol. 2009:301–51.

[18] Rockman MV (2012). The QTN program and the alleles that matter for evolution: all that's gold does not glitter. Evolution..

[19] Boyle EA, Li YI, Pritchard JK (2017). An expanded view of complex traits: from polygenic to Omnigenic. Cell..

[20] Pritchard JK, Di Rienzo A (2010). Adaptation - not by sweeps alone. Nat Rev Genet.

[21] Pavlidis P, Metzler D, Stephan W (2012). Selective sweeps in multilocus models of quantitative traits. Genetics..

[22] Chevin L-M, Hospital F (2008). Selective sweep at a quantitative trait locus in the presence of background genetic variation. Genetics..

[23] Jain K, Stephan W (2015). Response of Polygenic Traits Under Stabilizing Selection and Mutation When Loci Have Unequal Effects. G3&amp;#58; Genes|Genomes|Genetics.

[24] Tigano A, Friesen VL (2016). Genomics of local adaptation with gene flow. Mol Ecol.

[25] Remington DL (2015). Alleles versus mutations: understanding the evolution of genetic architecture requires a molecular perspective on allelic origins. Evolution..

[26] Yeaman S, Whitlock MC (2011). The genetic architecture of adaptation under migration-selection balance. Evolution..

[27] Joron M, Frezal L, Jones RT, Chamberlain NL, Lee SF, Haag CR (2011). Chromosomal rearrangements maintain a polymorphic supergene controlling butterfly mimicry. Nature..

[28] Jones FC, Grabherr MG, Chan YF, Russell P, Mauceli E, Johnson J (2012). The genomic basis of adaptive evolution in threespine sticklebacks. Nature..

[29] Samuk K, Owens GL, Delmore KE, Miller SE, Rennison DJ, Schluter D (2017). Gene flow and selection interact to promote adaptive divergence in regions of low recombination. Mol Ecol.

[30] Yeaman S (2015). Local adaptation by alleles of small effect. Am Nat.

[31] Kennish MJ, Olsson RK (1975). Effects of thermal discharges on the microstructural growth ofMercenaria mercenaria. Environ Geol.

[32] Hoagland K, Turner R (1980). Range extensions of teredinids (shipworms) and polychaetes in the vicinity of a temperate-zone nuclear generating station. Mar Biol.

[33] Mustard J, Carney M, Sen A (1999). The use of satellite data to quantify thermal effluent impacts. Estuar Coast Shelf Sci.

[34] Swanson C, Kim H-S, Sankaranarayanan S (2006). Modeling of temperature distributions in Mount Hope Bay due to thermal discharges from the Brayton Point Station. Northeastern Nat.

[35] O'Neill RJ, Englert TL, Ko JK (2006). Effects of Brayton Point Station's Thermal Discharge on Mount Hope Bay Winter Flounder. Northeastern Nat.

[36] DeAlteris JT, Englert TL, Burnett JAD (2006). Trends in Fish Abundance in Mount Hope Bay: Is the Brayton Point Power Station Affecting Fish Stocks?. Northeastern Nat.

[37] Calabretta CJ, Oviatt CA (2008). The response of benthic macrofauna to anthropogenic stress in Narragansett Bay, Rhode Island: a review of human stressors and assessment of community conditions. Mar Pollut Bull.

[38] Nyman L. Allelic Selection in a Fish(Gymnocephalus Cernua(L.)) Subjected to Hotwater Effluents. Fishery Board of Sweden Institute of Freshwater Res Report. 1975;(54):75–82.

[39] Nevo E, Shimony T, Libni M. Thermal selection of allozyme polymorphisms in barnacles. Nature. 1977;267(5613):699. 10.1038/267699a0 876389

[40] Yardley D, Avise JC, Gibbons JW, Smith MH, Gibbons J, Sharitz R (1974). Biochemical genetics of sunfish. III. Genetic subdivision of fish populations inhabiting heated waters.

[41] Mitton JB, Koehn RK (1975). Genetic organization and adaptive response of allozymes to ecological variables in Fundulus heteroclitus. Genetics..

[42] Smith MH, Smith MW, Scott SL, Liu EH, Jones JC. Rapid evolution in a post-thermal environment. Copeia. 1983:193–7.

[43] Porcelli D, Butlin RK, Gaston KJ, Joly D, Snook RR (2015). The environmental genomics of metazoan thermal adaptation. Heredity (Edinb).

[44] Menozzi P, Shi M, Lougarre A, Tang Z, Fournier D (2004). Mutations of acetylcholinesterase which confer insecticide resistance in Drosophila melanogaster populations. BMC Evol Biol.

[45] Oleksiak M, Karchner S, Jenny M, Franks D, Mark Welch D, Hahn M (2011). Transcriptomic assessment of resistance to effects of an aryl hydrocarbon receptor (AHR) agonist in embryos of Atlantic killifish (Fundulus heteroclitus) from a marine superfund site. BMC Genomics.

[46] Reid NM, Proestou DA, Clark BW, Warren WC, Colbourne JK, Shaw JR (2016). The genomic landscape of rapid repeated evolutionary adaptation to toxic pollution in wild fish. Science..

[47] Jensen JD. On the unfounded enthusiasm for soft selective sweeps. Nat Commun. 2014;5:5281-90. 10.1038/ncomms6281 25345443

[48] Hochachka PW, Somero GN (2001). Biochemical adaptation : mechanism and process in physiological evolution: mechanism and process in physiological evolution.

[49] Angilletta MJ (2009). Thermal adaptation: a theoretical and empirical synthesis: Oxford University press.

[50] Harmon JP, Moran NA, Ives AR (2009). Species response to environmental change: impacts of food web interactions and evolution. Science..

[51] Williams LM, Oleksiak MF (2008). Signatures of selection in natural populations adapted to chronic pollution. BMC Evol Biol.

[52] Meirmans PG (2012). The trouble with isolation by distance. Mol Ecol.

[53] Antao T, Lopes A, Lopes RJ, Beja-Pereira A, Luikart G (2008). LOSITAN: a workbench to detect molecular adaptation based on a Fst-outlier method. BMC Bioinformatics.

[54] Benjamini Y, Yekutieli D. The control of the false discovery rate in multiple testing under dependency. Ann Stat. 2001:1165–88.

[55] Narum SR (2006). Beyond Bonferroni: less conservative analyses for conservation genetics. Conserv Genet.

[56] Jombart T, Devillard S, Balloux F (2010). Discriminant analysis of principal components: a new method for the analysis of genetically structured populations. BMC Genet.

[57] Legendre P, Legendre LF. Numerical ecology. Amsterdam: Elsevier; 2012.

[58] Brommer JE (2011). Whither Pst? The approximation of Qst by Pst in evolutionary and conservation biology. J Evol Biol.

[59] Wilson BA, Petrov DA, Messer PW (2014). Soft selective sweeps in complex demographic scenarios. Genetics..

[60] Legendre P, Fortin MJ (2010). Comparison of the mantel test and alternative approaches for detecting complex multivariate relationships in the spatial analysis of genetic data. Mol Ecol Resour.

[61] Latch EK, Dharmarajan G, Glaubitz JC, Rhodes OE (2006). Relative performance of Bayesian clustering software for inferring population substructure and individual assignment at low levels of population differentiation. Conserv Genet.

[62] Gagnaire PA, Broquet T, Aurelle D, Viard F, Souissi A, Bonhomme F (2015). Using neutral, selected, and hitchhiker loci to assess connectivity of marine populations in the genomic era. Evol Appl.

[63] Xu S, Song N, Zhao L, Cai S, Han Z, Gao T (2017). Genomic evidence for local adaptation in the ovoviviparous marine fish Sebastiscus marmoratus with a background of population homogeneity. Sci Rep.

[64] Takahashi T, Sota T, Hori M (2013). Genetic basis of male colour dimorphism in a Lake Tanganyika cichlid fish. Mol Ecol.

[65] Milano I, Babbucci M, Cariani A, Atanassova M, Bekkevold D, Carvalho GR (2014). Outlier SNP markers reveal fine-scale genetic structuring across European hake populations (Merluccius merluccius). Mol Ecol.

[66] Fangue NA, Hofmeister M, Schulte PM (2006). Intraspecific variation in thermal tolerance and heat shock protein gene expression in common killifish, Fundulus heteroclitus. J Exp Biol.

[67] Teshima KM, Coop G, Przeworski M (2006). How reliable are empirical genomic scans for selective sweeps?. Genome Res.

[68] Lotterhos KE, Whitlock MC (2014). Evaluation of demographic history and neutral parameterization on the performance of FST outlier tests. Mol Ecol.

[69] Podrabsky JE, Somero GN (2004). Changes in gene expression associated with acclimation to constant temperatures and fluctuating daily temperatures in an annual killifish Austrofundulus limnaeus. J Exp Biol.

[70] Stephan W (2016). Signatures of positive selection: from selective sweeps at individual loci to subtle allele frequency changes in polygenic adaptation. Mol Ecol.

[71] Falconer DS, Mackay TFC. Introduction to quantitative genetics: Longman. Harlow: Prentice Hall; 1996.

[72] Hancock AM, Witonsky DB, Ehler E, Alkorta-Aranburu G, Beall C, Gebremedhin A (2010). Human adaptations to diet, subsistence, and ecoregion are due to subtle shifts in allele frequency. Proc Natl Acad Sci.

[73] Coop G, Pickrell JK, Novembre J, Kudaravalli S, Li J, Absher D (2009). The role of geography in human adaptation. PLoS Genet.

[74] Berg JJ, Coop G (2014). A population genetic signal of polygenic adaptation. PLoS Genet.

[75] Bergland AO, Behrman EL, O'Brien KR, Schmidt PS, Petrov DA (2014). Genomic evidence of rapid and stable adaptive oscillations over seasonal time scales in Drosophila. PLoS Genet.

[76] Shi H, Kichaev G, Pasaniuc B (2016). Contrasting the genetic architecture of 30 complex traits from summary association data. Am J Hum Genet.

[77] Laporte M, Pavey SA, Rougeux C, Pierron F, Lauzent M, Budzinski H (2016). RAD sequencing reveals within-generation polygenic selection in response to anthropogenic organic and metal contamination in North Atlantic eels. Mol Ecol.

[78] Schrider DR, Mendes FK, Hahn MW, Kern AD (2015). Soft shoulders ahead: spurious signatures of soft and partial selective sweeps result from linked hard sweeps. Genetics..

[79] Kaplan NL, Hudson R, Langley C (1989). The “hitchhiking effect” revisited. Genetics..

[80] Akey JM, Zhang G, Zhang K, Jin L, Shriver MD (2002). Interrogating a high-density SNP map for signatures of natural selection. Genome Res.

[81] Hohenlohe PA, Bassham S, Etter PD, Stiffler N, Johnson EA, Cresko WA (2010). Population genomics of parallel adaptation in threespine stickleback using sequenced RAD tags. PLoS Genet.

[82] Paaby AB, Bergland AO, Behrman EL, Schmidt PS (2014). A highly pleiotropic amino acid polymorphism in the Drosophila insulin receptor contributes to life-history adaptation. Evolution..

[83] Barrett RD, Schluter D (2008). Adaptation from standing genetic variation. Trends Ecol Evol.

[84] Powers DA, Place AR (1978). Biochemical genetics of Fundulus heteroclitus (L.). I. Temporal and spatial variation in gene frequencies of Ldh-B, Mdh-a, Gpi-B, and Pgm-a. Biochem Genet.

[85] Adams SM, Lindmeier JB, Duvernell DD (2006). Microsatellite analysis of the phylogeography, Pleistocene history and secondary contact hypotheses for the killifish, Fundulus heteroclitus. Mol Ecol.

[86] Prezeworski M, Coop G, Wall JD (2005). The signature of positive selection on standing genetic variation. Evolution..

[87] Hardie DC, Hebert PD (2004). Genome-size evolution in fishes. Can J Fish Aquat Sci.

[88] Lowry DB, Hoban S, Kelley JL, Lotterhos KE, Reed LK, Antolin MF (2017). Breaking RAD: an evaluation of the utility of restriction site-associated DNA sequencing for genome scans of adaptation. Mol Ecol Resour.

[89] Fitzpatrick B, Fordyce J, Niemiller M, Reynolds RG (2012). What can DNA tell us about biological invasions?. Biol Invasions.

[90] Kimura M (1980). Average time until fixation of a mutant allele in a finite population under continued mutation pressure: studies by analytical, numerical, and pseudo-sampling methods. Proc Natl Acad Sci.

[91] Lynch M (2010). Scaling expectations for the time to establishment of complex adaptations. Proc Natl Acad Sci.

[92] Reznick DN, Ghalambor CK (2001). The population ecology of contemporary adaptations: what empirical studies reveal about the conditions that promote adaptive evolution. Genetica..

[93] Reznick DNG, C.K. (2005). Selection in nature: experimental manipulations of natural populations. Integr Comp Biol.

[94] Irschick DJ, Meyers JJ, Husak JF, Le Galliard J (2008). How does selection operate on whole-organism functional performance capacities? A review and synthesis. Evol Ecol Res.

[95] Ivanova NV, Dewaard JR, Hebert PDN (2006). An inexpensive, automation-friendly protocol for recovering high-quality DNA. Mol Ecol Notes.

[96] Elshire RJ, Glaubitz JC, Sun Q, Poland JA, Kawamoto K, Buckler ES (2011). A robust, simple genotyping-by-sequencing (GBS) approach for high diversity species. PLoS One.

[97] Bradbury PJ, Zhang Z, Kroon DE, Casstevens TM, Ramdoss Y, Buckler ES (2007). TASSEL: software for association mapping of complex traits in diverse samples. Bioinformatics..

[98] Glaubitz JC, Casstevens TM, Lu F, Harriman J, Elshire RJ, Sun Q (2014). TASSEL-GBS: a high capacity genotyping by sequencing analysis pipeline. PLoS One.

[99] Hosking L, Lumsden S, Lewis K, Yeo A, McCarthy L, Bansal A (2004). Detection of genotyping errors by hardy-Weinberg equilibrium testing. Eur J Hum Genet.

[100] Teo YY, Fry AE, Clark TG, Tai ES, Seielstad M (2007). On the usage of HWE for identifying genotyping errors. Ann Hum Genet.

[101] Excoffier L, Laval G, Schneider S (2005). Arlequin (version 3.0): an integrated software package for population genetics data analysis. Evol Bioinformatics Online.

[102] Beaumont MA, Nichols RA. Evaluating Loci for Use in the Genetic Analysis of Population Structure 1996 1996-12-22 00:00:00. 1619–26 p.

[103] Chessel D, Dufour AB, Thioulouse J (2004). The ade4 package-I-one-table methods. R News.

[104] Hurvich CM, Simonoff JS, Tsai CL (1998). Smoothing parameter selection in nonparametric regression using an improved Akaike information criterion. J R Stat Soc.

[105] Falush D, Stephens M, Pritchard JK (2003). Inference of population structure using multilocus genotype data: linked loci and correlated allele frequencies. Genetics..

[106] Evanno G, Regnaut S, Goudet J (2005). Detecting the number of clusters of individuals using the software STRUCTURE: a simulation study. Mol Ecol.

[107] Kopelman NM, Mayzel J, Jakobsson M, Rosenberg NA, Mayrose I. CLUMPAK: a program for identifying clustering modes and packaging population structure inferences across K. Mol Ecol Resour. 2015;15(5):1179-91. 10.1111/1755-0998.12387 PMC4534335 25684545

[108] Yeomans KA, Golder PA. The Guttman-Kaiser criterion as a predictor of the number of common factors. Statistician. 1982:221–9.

[109] Borcard D, Legendre P (2002). All-scale spatial analysis of ecological data by means of principal coordinates of neighbour matrices. Ecol Model.

[110] Oksanen J, Kindt R, Legendre P, O’Hara B, Stevens MHH, Oksanen MJ (2007). The vegan package. Community Ecol Package.

[111] Dray S, Blanchet G, Borcard D, Guenard G, Jombart T, Larocque G, et al. Adespatial: Multivariate Multiscale Spatial Analysis. R package version 0.0–4. 2016.

[112] Borcard D, Legendre P, Drapeau P (1992). Partialling out the spatial component of ecological variation. Ecology..

[113] McVean G (2009). A genealogical interpretation of principal components analysis. PLoS Genet.

[114] Meirmans PG (2015). Seven common mistakes in population genetics and how to avoid them. Mol Ecol.

[115] Huang DW, Sherman BT, Lempicki RA (2008). Systematic and integrative analysis of large gene lists using DAVID bioinformatics resources. Nat Protocols.

[116] Lutterschmidt WI, Hutchison VH (1997). The critical thermal maximum: history and critique. Can J Zool.

[117] Dayan DI, Du X, Baris TZ, Wagner DN, Crawford DL, Oleksiak MF. Data from: population genomics of rapid evolution in natural populations: polygenic selection in response to power station thermal effluents. Dryad Data Repository. 10.5061/dryad.3503s21. 10.1186/s12862-019-1392-5 PMC6390305 30808292

