# Supplementary figures and images for: Population genomics of rapid evolution in natural populations: polygenic selection in response to power station thermal effluents
Source: BMC Evol Biol. 2019 Feb 26;19:61. doi: 10.1186/s12862-019-1392-5 (PMC6390305; doi:10.1186/s12862-019-1392-5)

**(A)**

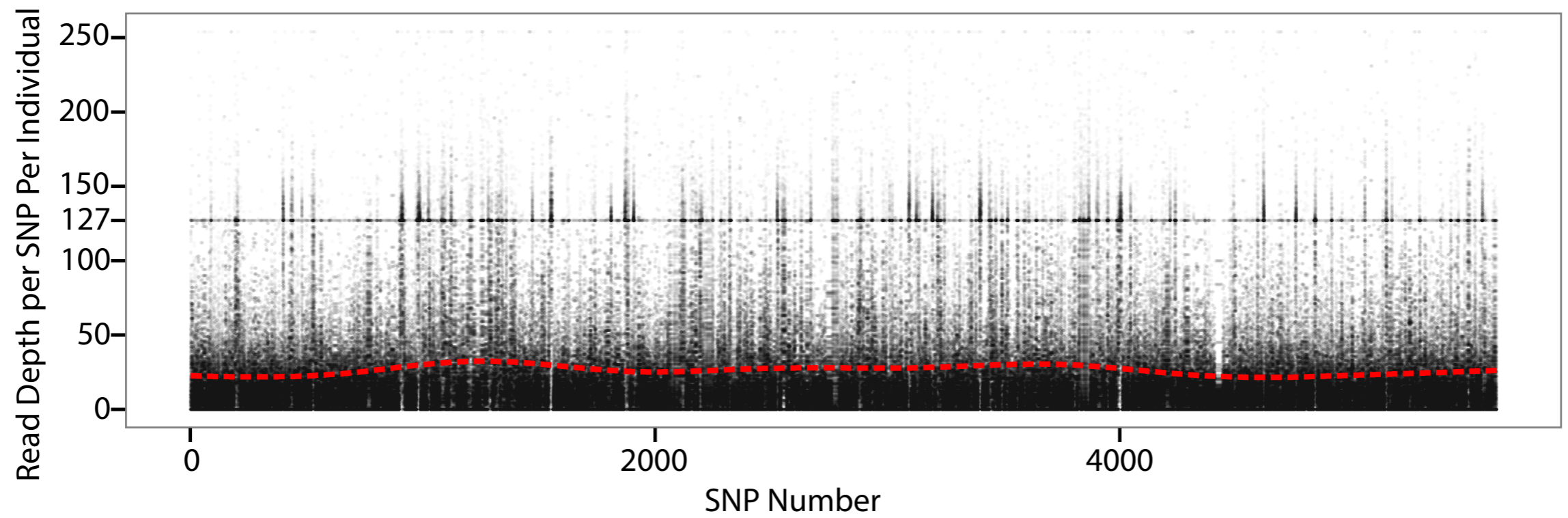

**(B)**

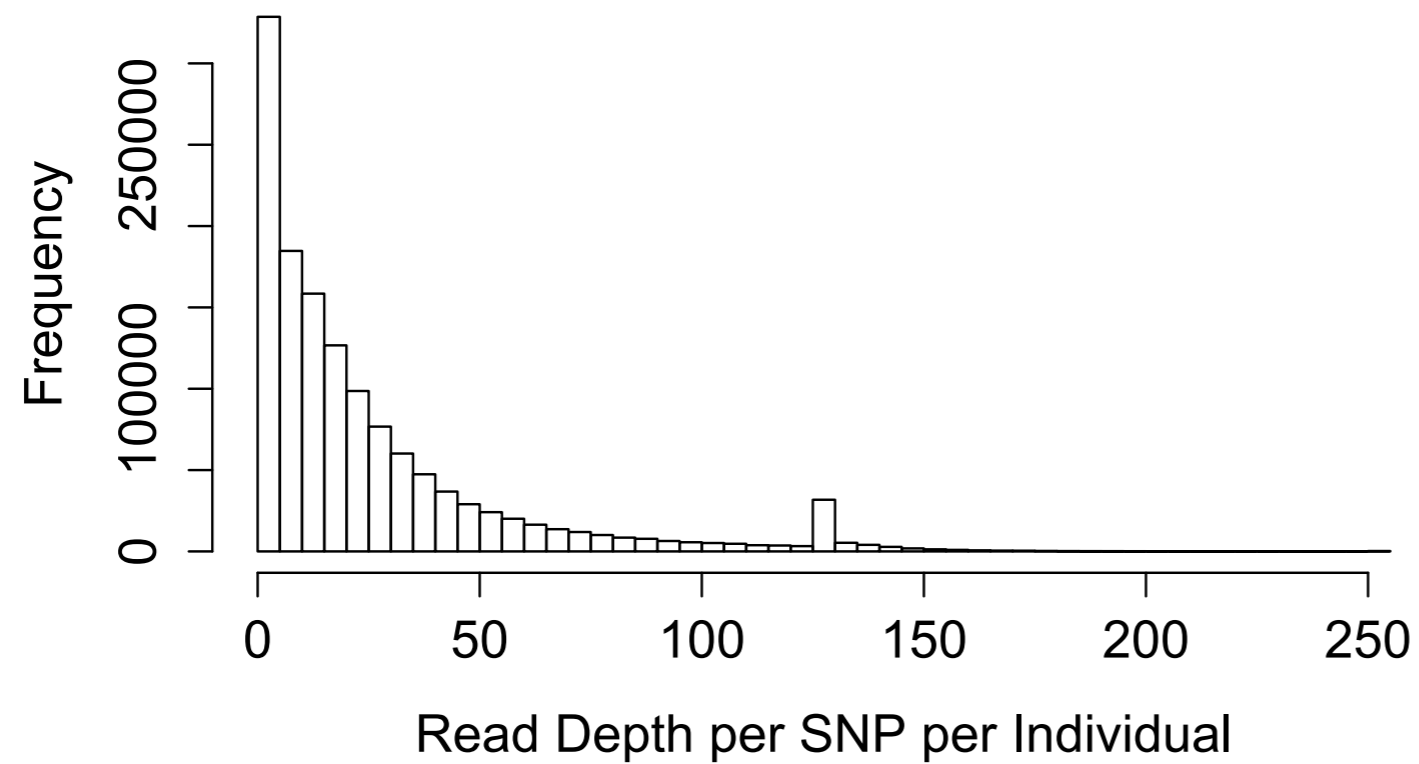

Supplement: Supplementary file 1 — Figure S1. Depth of read per individual at each SNP in the 5.4 k SNP dataset. (A) Mean depth across individuals represented by red line. (B) Frequency histogram of reads per SNP per individual. (PDF 1857 kb) [file 12862_2019_1392_MOESM1_ESM.pdf]

(A)

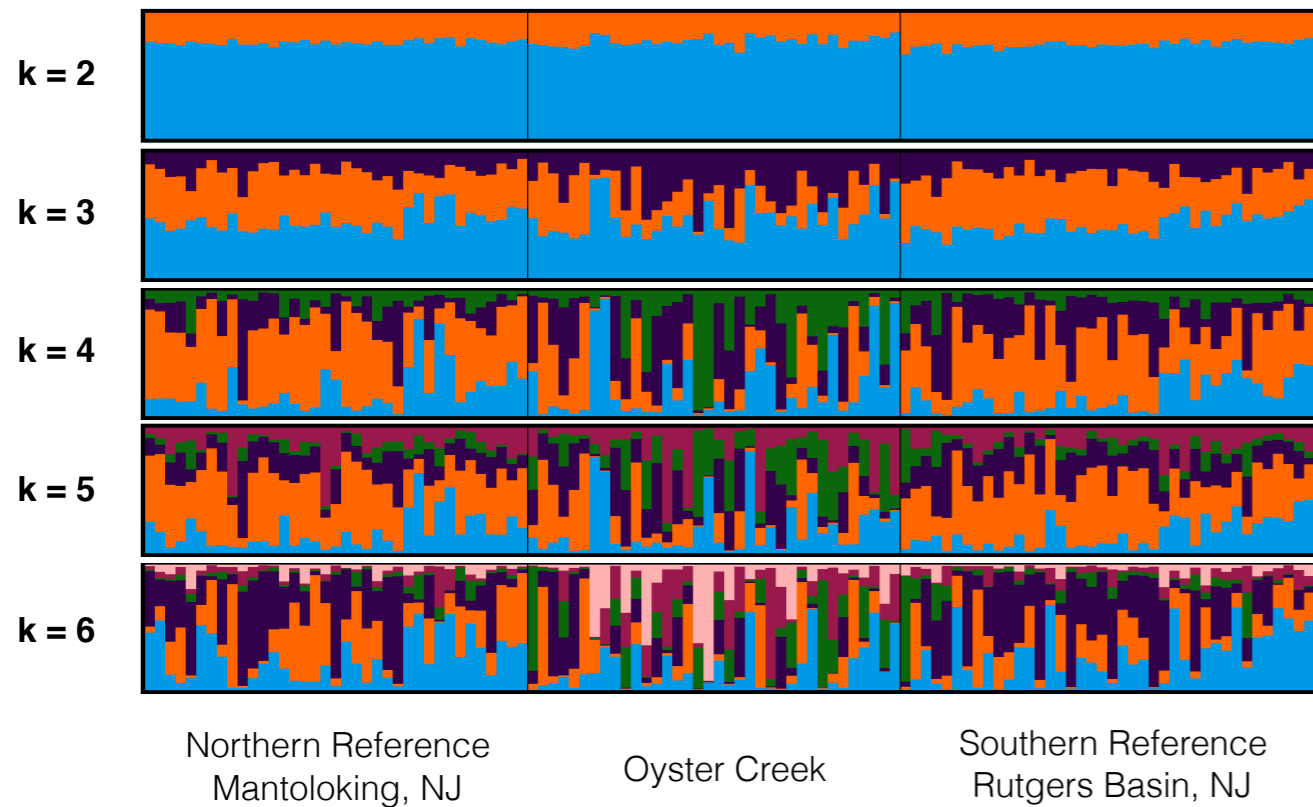

(B)

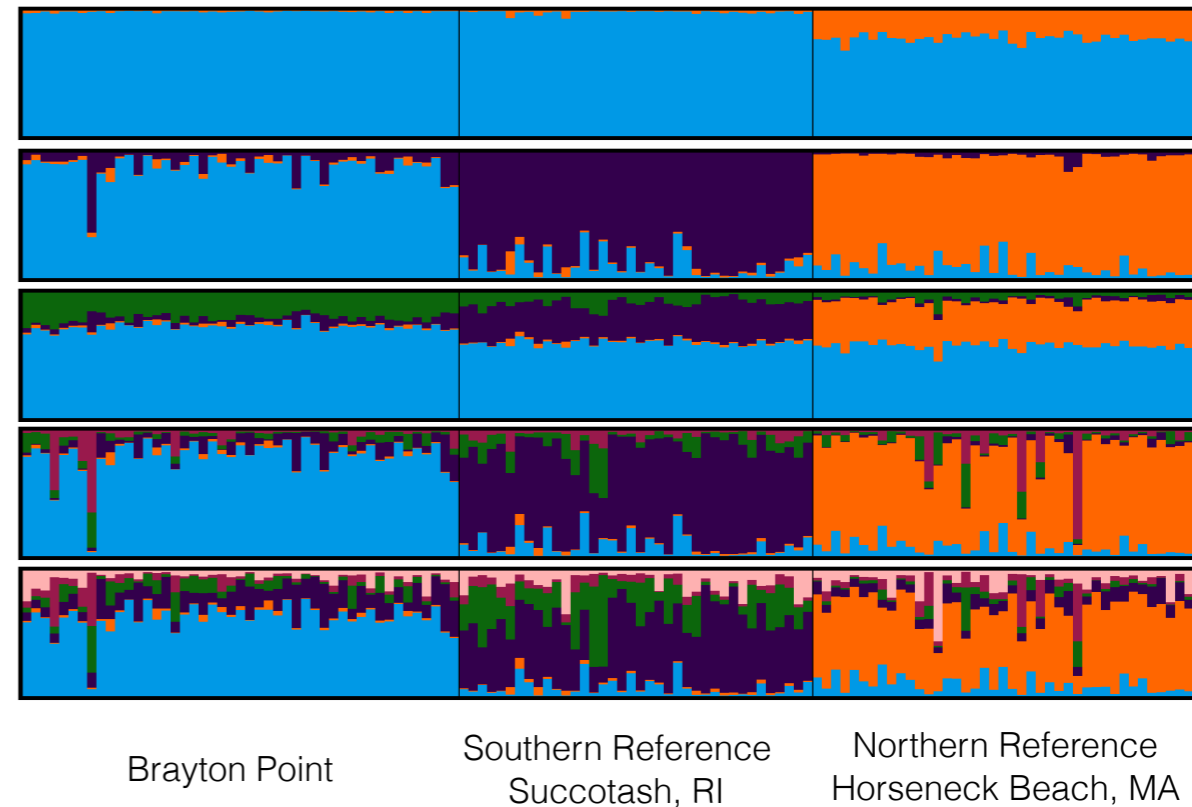

Supplement: Supplementary file 3 — Figure S8. STRUCTURE results for after LD thinning the full dataset. STRUCTURE plots for LD thinned dataset (r2 < 0.5, 3992 SNPs) among (A) Brayton Point triad, best k = 3, and (B) Oyster Creek triad, best k = 1. (PDF 1589 kb) [file 12862_2019_1392_MOESM3_ESM.pdf]

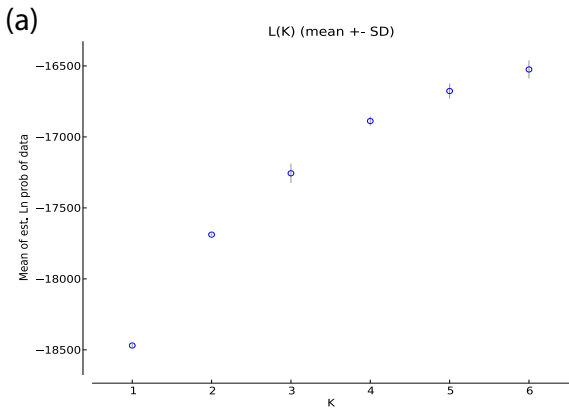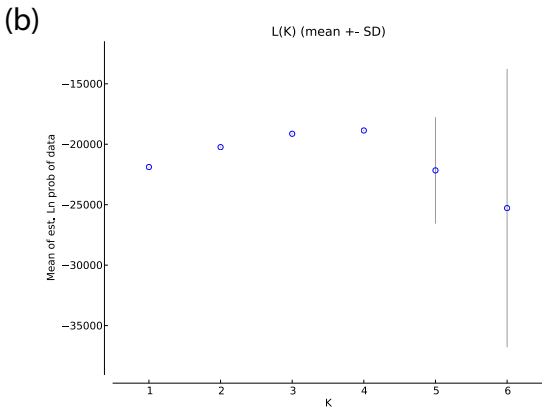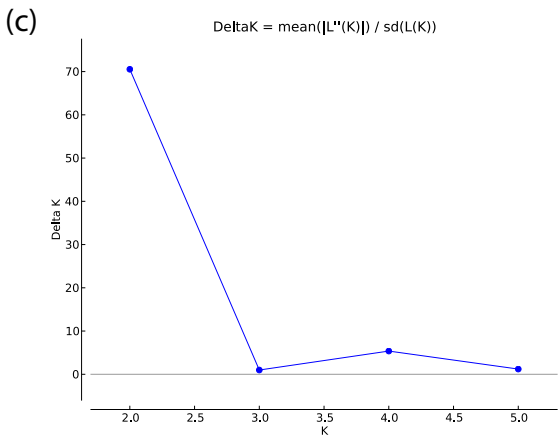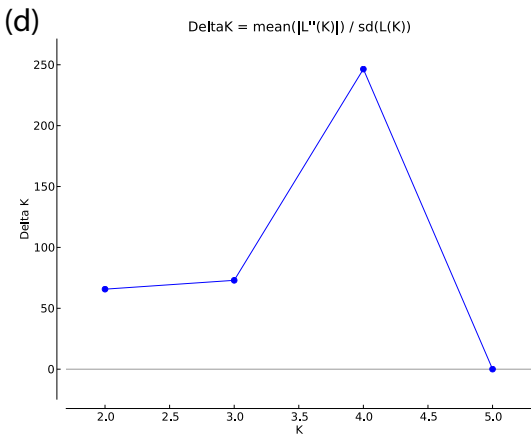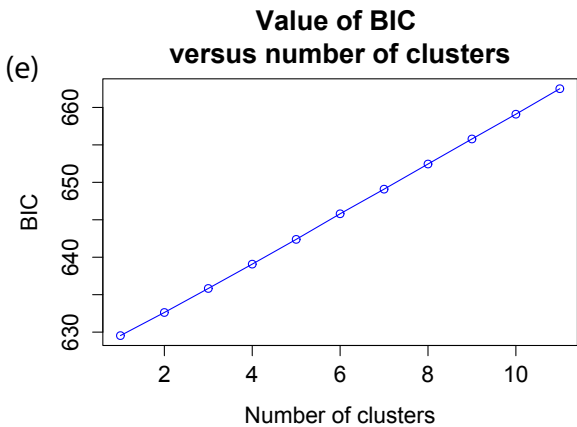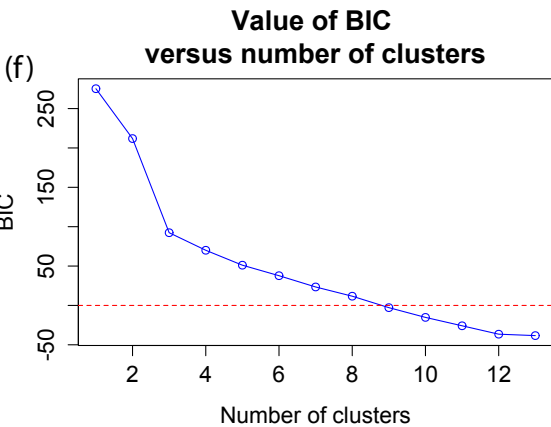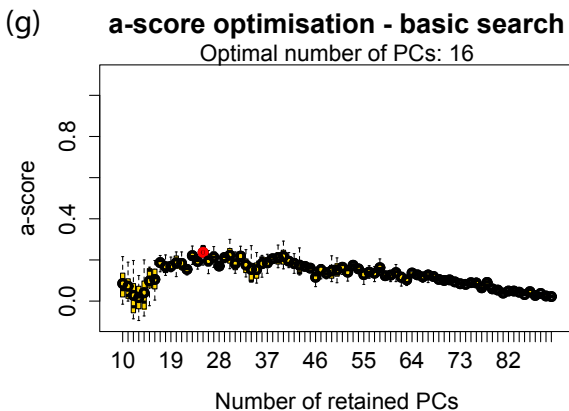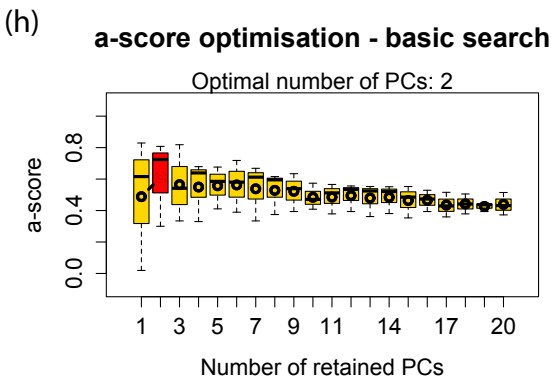

Supplement: Supplementary file 4 — Figure S3. Supporting population genetic structure data. (PDF 820 kb) [file 12862_2019_1392_MOESM4_ESM.pdf]

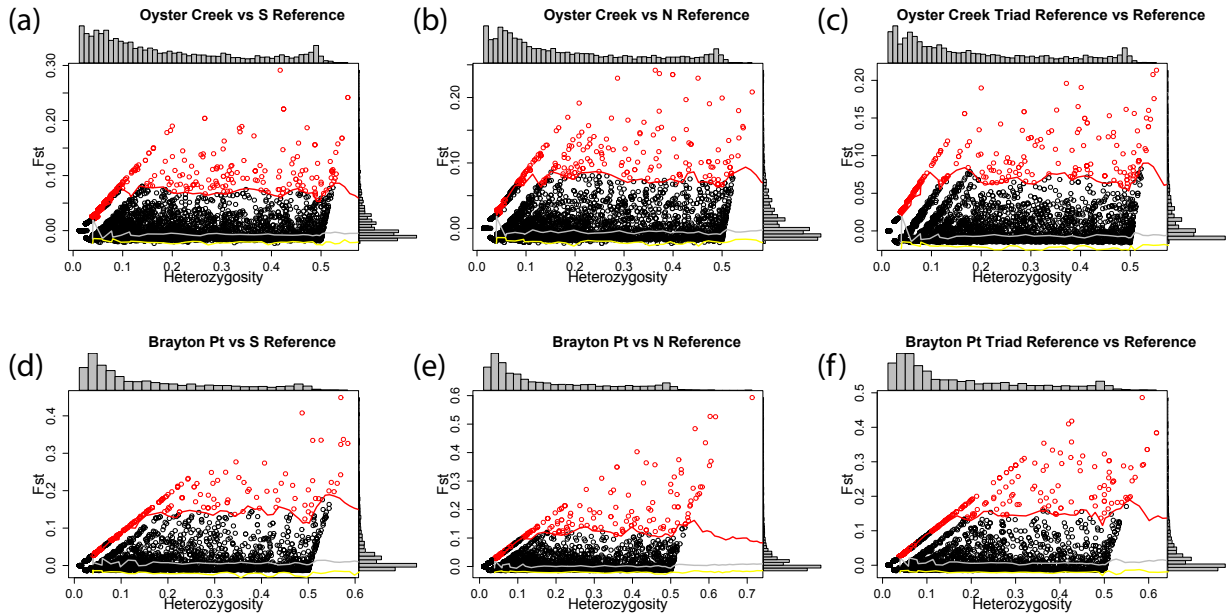

Supplement: Supplementary file 5 — Figure S2. Outlier Scans. Lositan output with distributions of FST values and observed H for all within triad pairwise comparisons. (PDF 1080 kb) [file 12862_2019_1392_MOESM5_ESM.pdf]

(A)

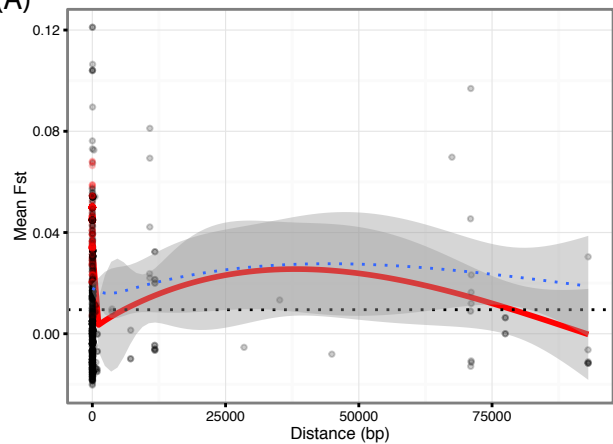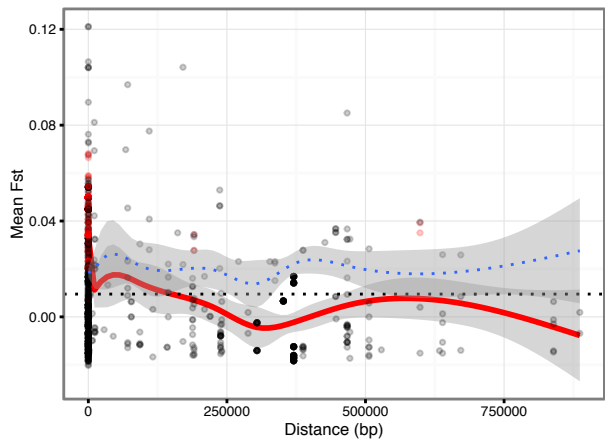

(B)

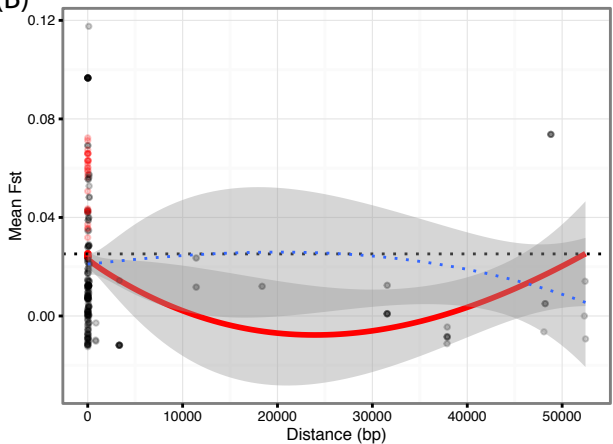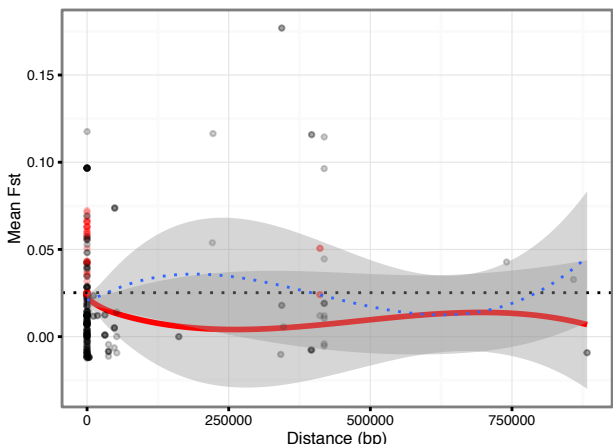

Supplement: Supplementary file 7 — Figure S5. Decay of FST away from candidate SNPs. Mean reference vs. effluent FST values at SNPs with 1mb and 100 kb from candidate SNPs for the Oyster Creek triad (a) and Brayton Point triad (b). Distance is presented in base pairs from candidate SNP, smoothing line is the Loess-smoothed mean FST value with 95% confidence intervals, dashed line is the mean genome-wide FST value estimate (dashed black line) for both reference vs effluent comparisons within the triad, dashed smoothing line (blue) is Loess-smoothed mean of a random permutation of distance vs. mean reference vs. effluent FST values, red SNPs are candidate loci. (PDF 3502 kb) [file 12862_2019_1392_MOESM7_ESM.pdf]

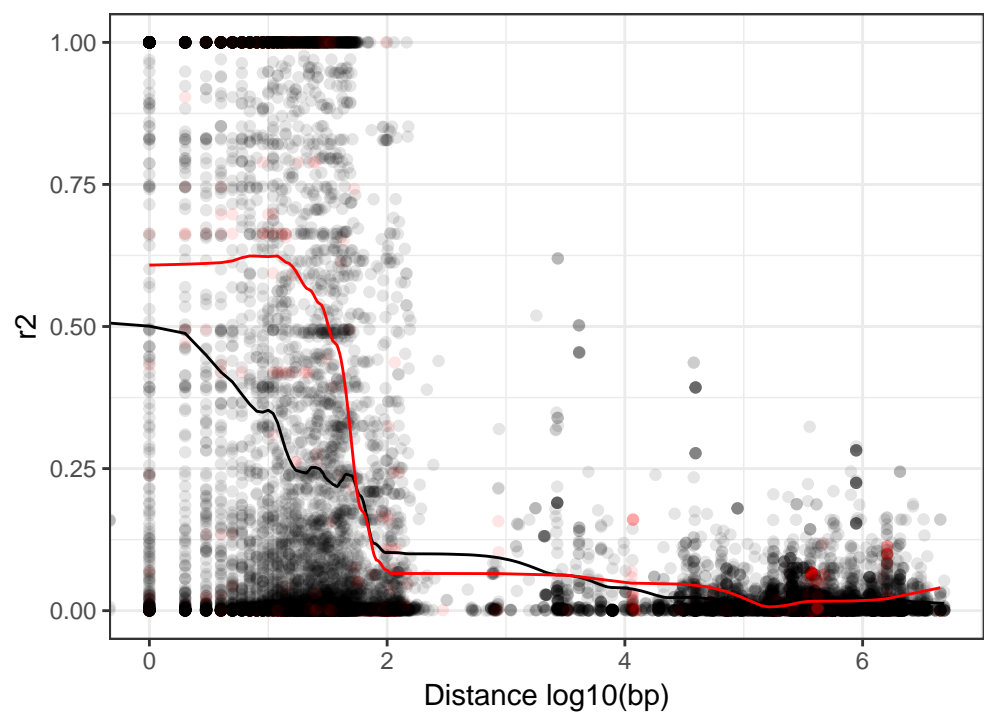

Supplement: Supplementary file 8 — Figure S4. Long Range LD Decay. R2 among single SNP pairs across the full dataset, with loess smoothing line for SNP pairs that contain a candidate (red) and those that do not (black). (PDF 649 kb) [file 12862_2019_1392_MOESM8_ESM.pdf]

# Pst variations: Probe

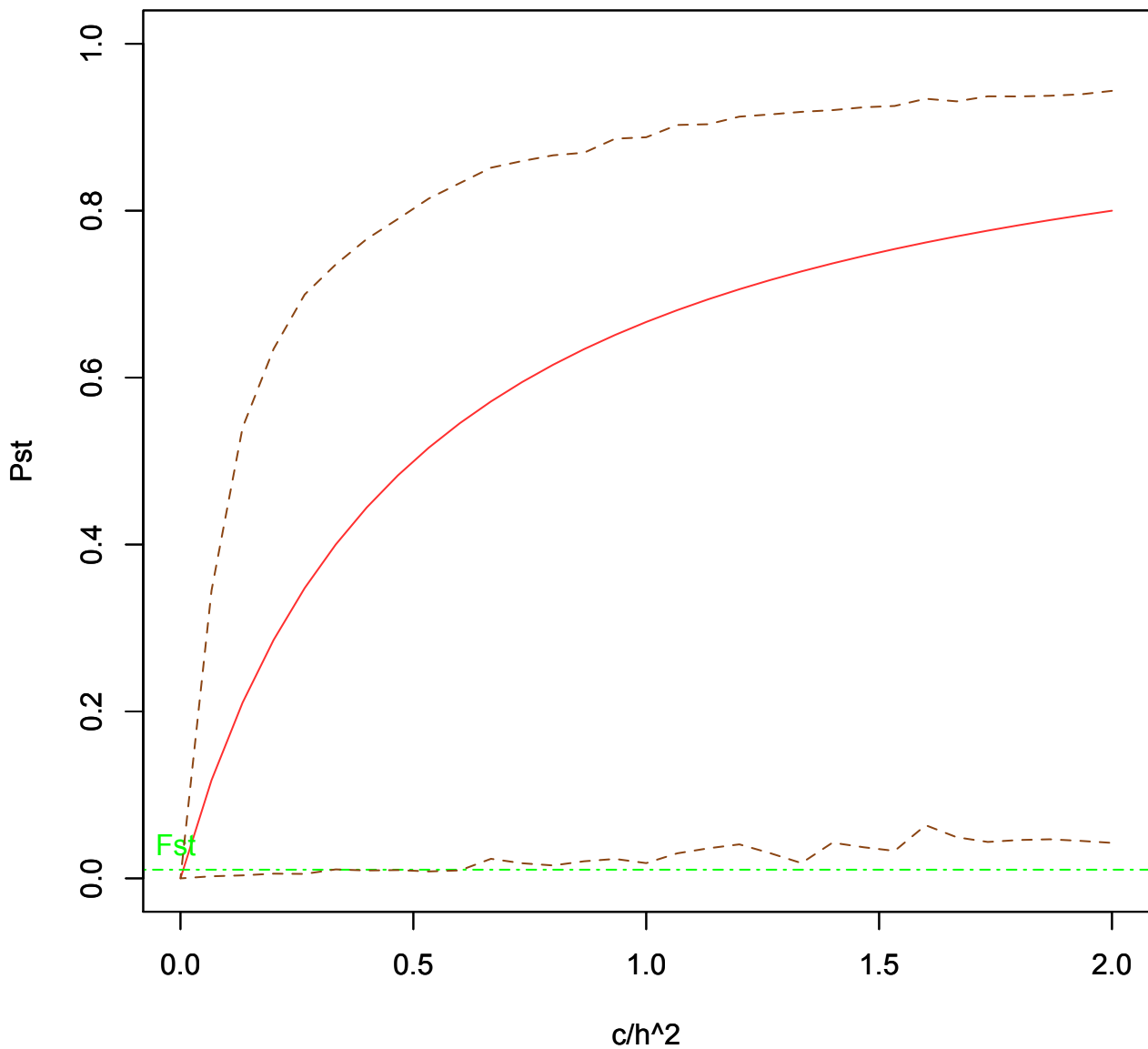

Supplement: Supplementary file 10 — Figure S7. PST-FST Comparison. PST and it’s 95% confidence interval for critical thermal maximum differences among F. heteroclitus from Oyster Creek and its northern reference population. PST is estimated for a range of c/h2, where c is proportion of the total variance that is presumed to be because of additive genetic effects across populations and h2 is narrow sense heritability. FST is plotted in green. PST exceeds FST for the majority of the parameter space, suggesting that the degree of phenotypic divergence exceeds that expected given the extent of genome-wide divergence alone. (PDF 194 kb) [file 12862_2019_1392_MOESM10_ESM.pdf]

(A)

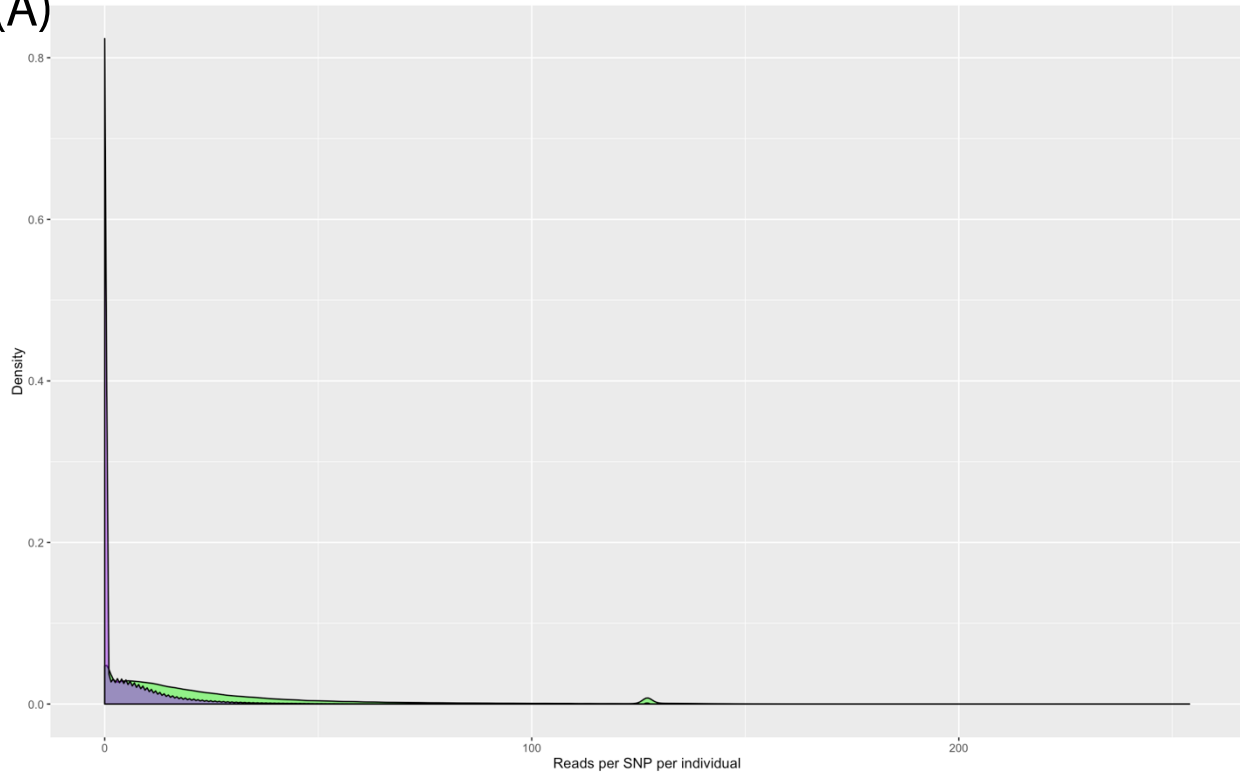

(B)

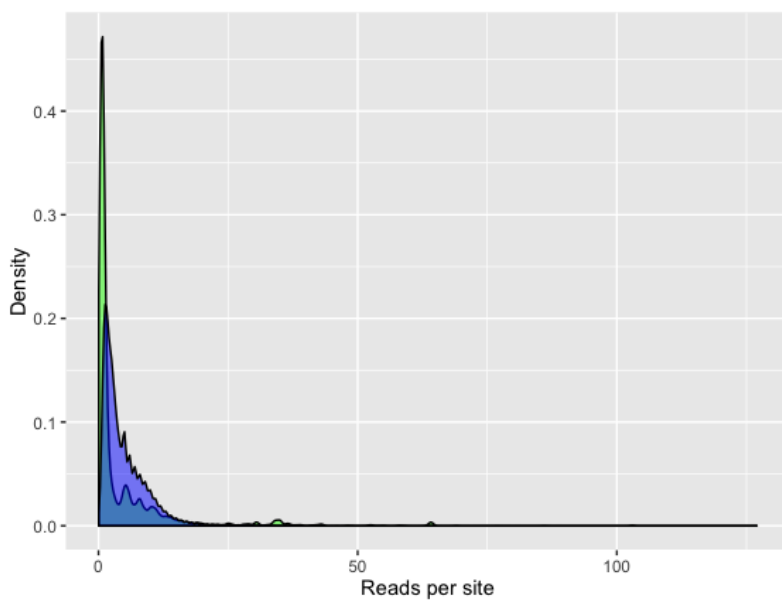

(C)

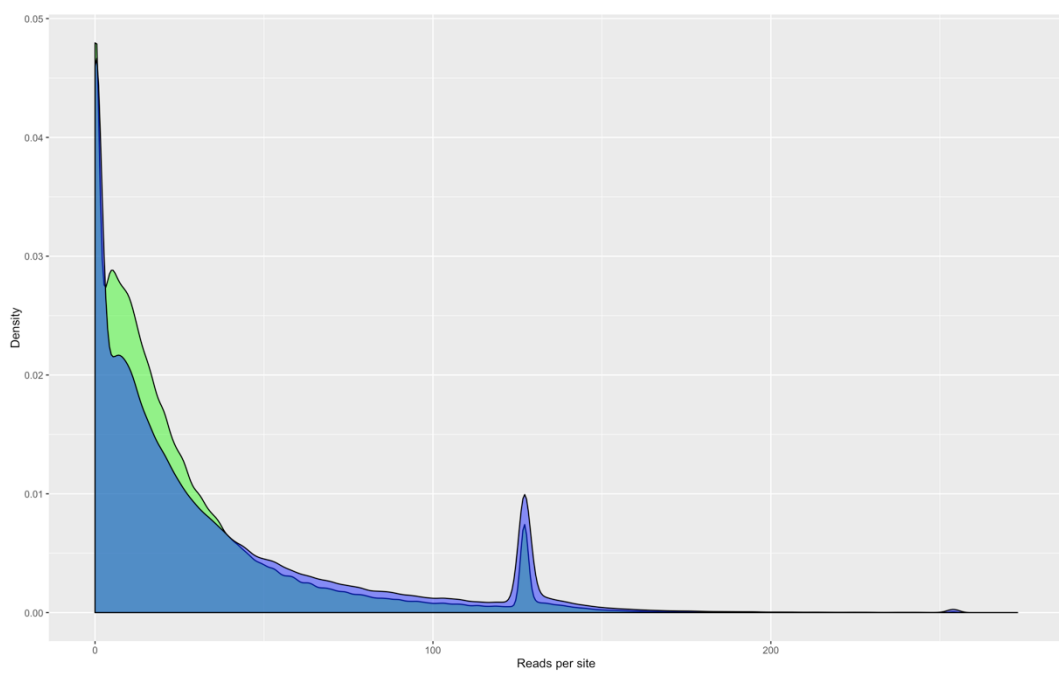

Supplement: Supplementary file 11 — Figure S6. Filtering impacts on coverage summary. (A) Density plot of reads per SNP per individual for the filtered (green) and raw (purple) datasets. (B) Density plot of non-zero reads per unique tag per individual for filtered dataset (purple) and the raw dataset (green). (C) Density plot of reads per SNP per individual for the filtered (green) dataset and the raw dataset excluding with SNPs where > 75% of individuals demonstrate zero reads (blue). Summary: There are large differences in coverage between our filtered (5.4 k) and raw SNP (314 k) datasets. The median read depth per SNP per individual of the raw dataset (panel A, purple) is 0 (filtered data median = 16, green). The mean read depth per SNP per individual of the raw dataset is ~ 4.4 (filtered data mean = ~ 26.3). While we base our filtering on missingness across SNPs and individuals (missing genotype calls results from less than 5 reads per unique 64 bp sequence tag within an individual), the majority of SNPs we filter out have zero reads in the majority of individuals, but coverage similar to our filtered dataset in the remaining individuals (panel B and C). (PDF 746 kb) [file 12862_2019_1392_MOESM11_ESM.pdf]
