# Supplementary material for: Population genomics of rapid evolution in natural populations: polygenic selection in response to power station thermal effluents
Source: BMC Evol Biol. 2019 Feb 26;19:61. doi: 10.1186/s12862-019-1392-5 (PMC6390305; doi:10.1186/s12862-019-1392-5)
Supplement: Supplementary file 2 — Table S1. AMOVA results for neutral dataset. (DOCX 38 kb) [file 12862_2019_1392_MOESM2_ESM.docx]

Supplemental table 1

Table 2: AMOVA Results using neutral dataset

| Variance Component | df | % variation | Φ Statistic | P |
| --- | --- | --- | --- | --- |
| Among triads | 1 | 5.73 | Φ_CT_ = 0.05725 | p = 0.099 |
| Among populations within triads | 4 | 0.70 | Φ_SC_ =0.00741* | p < 0.00001 |
| Among individuals within populations | 472 | 93.58 | Φ_ST_ =0.06424* | p < 0.00001 |
| Total | 477 |  |  |  |
