# Supplementary material for: Population genomics of rapid evolution in natural populations: polygenic selection in response to power station thermal effluents
Source: BMC Evol Biol. 2019 Feb 26;19:61. doi: 10.1186/s12862-019-1392-5 (PMC6390305; doi:10.1186/s12862-019-1392-5)
Supplement: Supplementary file 6 — Figure S9. STRUCTURE results for after LD thinning the outlier dataset. STRUCTURE plots at best k for the most differentiated SNPs, LD thinned (r2 < 0.5) among (A) Brayton Point and (B) Oyster Creek triads. (PDF 1141 kb) [file 12862_2019_1392_MOESM6_ESM.pdf]

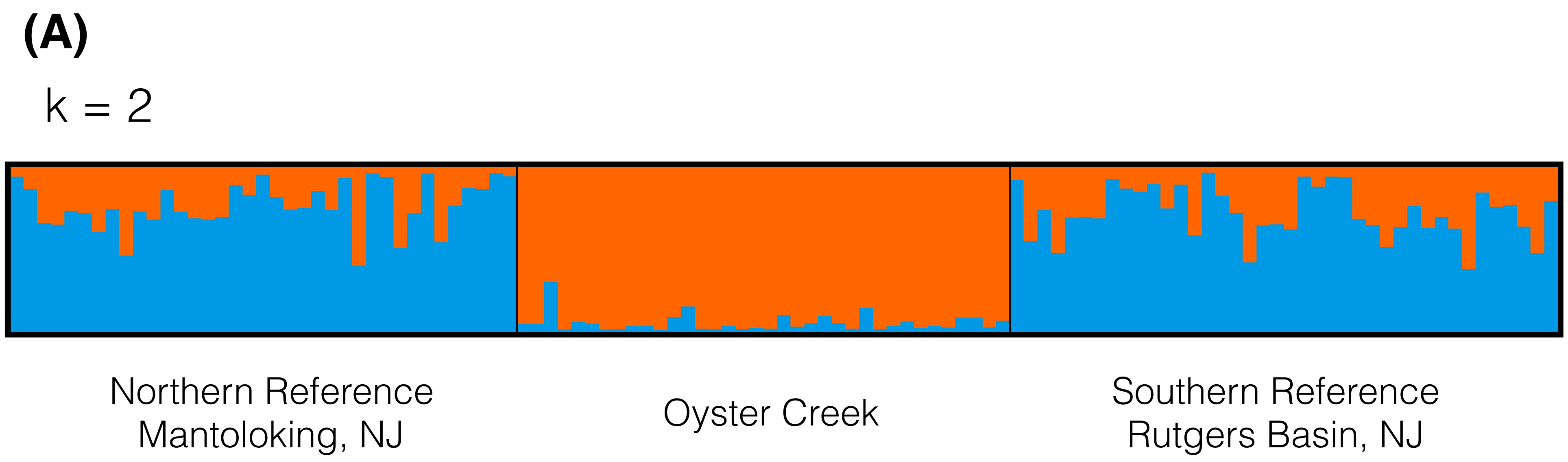
(A)
k = 2
Northern Reference
Mantoloking, NJ
Oyster Creek
Southern Reference
Rutgers Basin, NJ

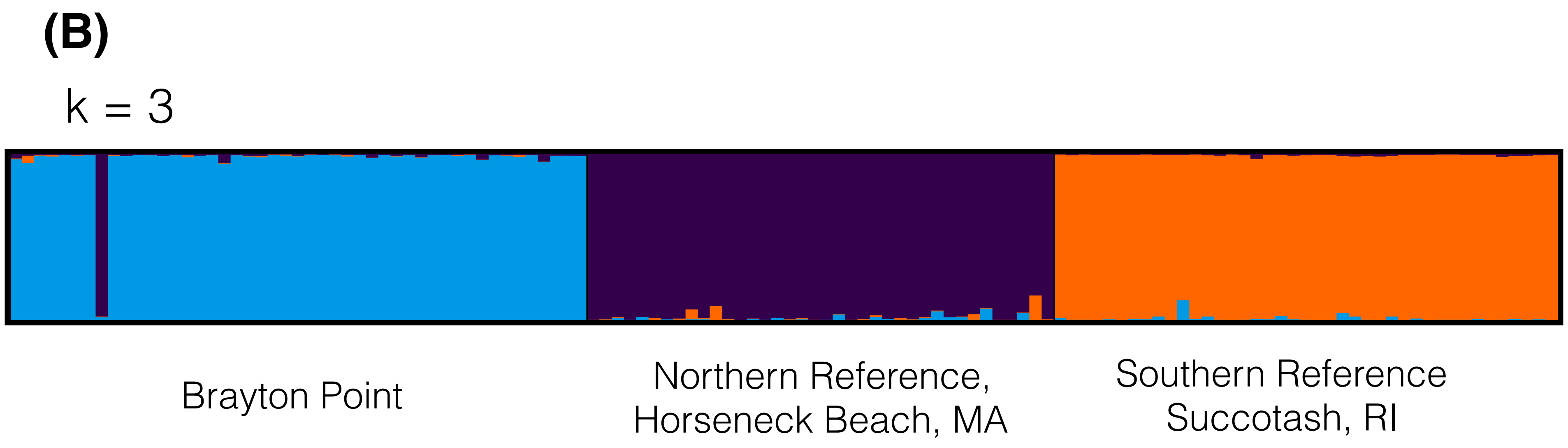
(B)
k = 3
Brayton Point
Northern Reference,
Horseneck Beach, MA
Southern Reference
Succotash, RI
