# Supplementary material for: Population genomics of rapid evolution in natural populations: polygenic selection in response to power station thermal effluents
Source: BMC Evol Biol. 2019 Feb 26;19:61. doi: 10.1186/s12862-019-1392-5 (PMC6390305; doi:10.1186/s12862-019-1392-5)
Supplement: Supplementary file 9 — Table S2. Functional annotation clusters for Oyster Creek (top) and Brayton Point (below). For each cluster the EASE score, functional annotation terms, gene identifiers, p-value for individual annotation terms, and fold enrichment relative to the F. heteroclitus background are presented. (DOCX 26 kb) [file 12862_2019_1392_MOESM9_ESM.docx]

| **Oyster Creek** |  |  |  | |  |  |
| --- | --- | --- | --- | --- | --- | --- |
| **Annotation Cluster 1** | **Enrichment Score: 1.452902979843862** | | | | |  |
| Term | Count | PValue | Genes | Fold Enrichment | | |
| GO:0070271~protein complex biogenesis | 5 | 0.02332372 | Q12846, Q8WZ74, Q06828, Q86YH6, Q86UR5 | | 4.34551887 |  |
| GO:0006461~protein complex assembly | 5 | 0.02332372 | Q12846, Q8WZ74, Q06828, Q86YH6, Q86UR5 | | 4.34551887 |  |
| GO:0065003~macromolecular complex assembly | 5 | 0.0481087 | Q12846, Q8WZ74, Q06828, Q86YH6, Q86UR5 | | 3.45684803 |  |
| GO:0043933~macromolecular complex subunit organization | 5 | 0.05896156 | Q12846, Q8WZ74, Q06828, Q86YH6, Q86UR5 | | 3.23245614 |  |
|  |  |  |  | |  |  |
| **Annotation Cluster 2** | **Enrichment Score: 1.2499423763407722** | | | | |  |
| Term | Count | PValue | Genes | | Fold Enrichment |  |
| repeat:3 | 3 | 0.04479619 | P56159, P19835, Q9UBC2 | | 8.66859717 |  |
| repeat:1 | 3 | 0.06260854 | P56159, P19835, Q9UBC2 | | 7.18070362 |  |
| repeat:2 | 3 | 0.06343052 | P56159, P19835, Q9UBC2 | | 7.12751323 |  |
|  |  |  |  | |  |  |
| **Annotation Cluster 3** | **Enrichment Score: 1.2380244993815794** | | | | |  |
| Term | Count | PValue | Genes | | Fold Enrichment |  |
| GO:0007268~synaptic transmission | 4 | 0.02230305 | Q12846, Q99996, Q8WZ74, Q86UR5 | | 6.32618026 |  |
| GO:0019226~transmission of nerve impulse | 4 | 0.03512635 | Q12846, Q99996, Q8WZ74, Q86UR5 | | 5.30215827 |  |
| GO:0050877~neurological system process | 4 | 0.24656374 | Q12846, Q99996, Q8WZ74, Q86UR5 | | 2.23672231 |  |
|  |  |  |  | |  |  |
| **Annotation Cluster 4** | **Enrichment Score: 0.8445345912345504** | | | | |  |
| Term | Count | PValue | Genes | | Fold Enrichment |  |
| domain:Ig-like C2-type 1 | 3 | 0.06016516 | Q8NFZ8, Q13203, P40199 | | 7.34514722 |  |
| domain:Ig-like C2-type 2 | 3 | 0.0617903 | Q8NFZ8, Q13203, P40199 | | 7.23469388 |  |
| IPR003598:Immunoglobulin subtype 2 | 3 | 0.08812145 | Q8NFZ8, Q13203, P40199 | | 5.8687472 |  |
| SM00408:IGc2 | 3 | 0.11917352 | Q8NFZ8, Q13203, P40199 | | 4.81922652 |  |
| IPR003599:Immunoglobulin subtype | 3 | 0.16354805 | Q8NFZ8, Q13203, P40199 | | 4.02364865 |  |
| SM00409:IG | 3 | 0.21613796 | Q8NFZ8, Q13203, P40199 | | 3.30409091 |  |
| Immunoglobulin domain | 3 | 0.24215297 | Q8NFZ8, Q13203, P40199 | | 3.11291038 |  |
| IPR007110:Immunoglobulin-like | 3 | 0.26531442 | Q8NFZ8, Q13203, P40199 | | 2.91025546 |  |
| IPR013783:Immunoglobulin-like fold | 3 | 0.28277096 | Q8NFZ8, Q13203, P40199 | | 2.78074147 |  |
|  |  |  |  | |  |  |
| **Annotation Cluster 5** | **Enrichment Score: 0.6614410900036688** | | | | |  |
| Term | Count | PValue | Genes | | Fold Enrichment |  |
| GO:0006887~exocytosis | 3 | 0.02151895 | Q12846, O00471, Q86UR5 | | 12.7068966 |  |
| GO:0032940~secretion by cell | 3 | 0.06586477 | Q12846, O00471, Q86UR5 | | 6.86645963 |  |
| GO:0046907~intracellular transport | 3 | 0.4460397 | Q12846, O00471, Q86UR5 | | 1.93269231 |  |
| GO:0015031~protein transport | 3 | 0.52776716 | Q12846, O00471, Q86UR5 | | 1.65990991 |  |
| GO:0045184~establishment of protein localization | 3 | 0.53354792 | Q12846, O00471, Q86UR5 | | 1.64264487 |  |
| GO:0008104~protein localization | 3 | 0.60379543 | Q12846, O00471, Q86UR5 | | 1.44888598 |  |
|  |  |  |  | |  |  |
| **Annotation Cluster 6** | **Enrichment Score: 0.5973339694303236** | | | | |  |
| Term | Count | PValue | Genes | | Fold Enrichment |  |
| GO:0004672~protein kinase activity | 4 | 0.14118682 | Q99996, Q8WZ74, O94921, Q00532 | | 2.92984262 |  |
| GO:0006468~protein amino acid phosphorylation | 4 | 0.1864262 | Q99996, Q8WZ74, O94921, Q00532 | | 2.57692308 |  |
| GO:0016310~phosphorylation | 4 | 0.27172093 | Q99996, Q8WZ74, O94921, Q00532 | | 2.12391931 |  |
| GO:0006793~phosphorus metabolic process | 4 | 0.37971082 | Q99996, Q8WZ74, O94921, Q00532 | | 1.75267539 |  |
| GO:0006796~phosphate metabolic process | 4 | 0.37971082 | Q99996, Q8WZ74, O94921, Q00532 | | 1.75267539 |  |
|  |  |  |  | |  |  |
| **Annotation Cluster 7** | **Enrichment Score: 0.5552302318226242** | | | | |  |
| Term | Count | PValue | Genes | | Fold Enrichment |  |
| IPR008271:Serine/threonine protein kinase, active site | 3 | 0.20818687 | Q99996, O94921, Q00532 | | 3.4376804 |  |
| IPR017442:Serine/threonine protein kinase-related | 3 | 0.21323832 | Q99996, O94921, Q00532 | | 3.38294027 |  |
| GO:0004674~protein serine/threonine kinase activity | 3 | 0.24213516 | Q99996, O94921, Q00532 | | 3.06461538 |  |
| serine/threonine-protein kinase | 3 | 0.25520273 | Q99996, O94921, Q00532 | | 3.00106929 |  |
| IPR017441:Protein kinase, ATP binding site | 3 | 0.28995625 | Q99996, O94921, Q00532 | | 2.73070242 |  |
| IPR000719:Protein kinase, core | 3 | 0.30840304 | Q99996, O94921, Q00532 | | 2.60993426 |  |
| kinase | 3 | 0.52926781 | Q99996, O94921, Q00532 | | 1.66781555 |  |
|  |  |  |  | |  |  |
| **Annotation Cluster 8** | **Enrichment Score: 0.4446996603551917** | | | | |  |
| Term | Count | PValue | Genes | | Fold Enrichment |  |
| GO:0045934~negative regulation of nucleobase, nucleoside, nucleotide and nucleic acid metabolic process | 3 | 0.31214765 | Q14865, O75943, O43251 | | 2.5649652 |  |
| GO:0051172~negative regulation of nitrogen compound metabolic process | 3 | 0.31704122 | Q14865, O75943, O43251 | | 2.53555046 |  |
| GO:0010558~negative regulation of macromolecule biosynthetic process | 3 | 0.34335006 | Q14865, O75943, O43251 | | 2.38768898 |  |
| GO:0031327~negative regulation of cellular biosynthetic process | 3 | 0.35303136 | Q14865, O75943, O43251 | | 2.3372093 |  |
| GO:0009890~negative regulation of biosynthetic process | 3 | 0.36363308 | Q14865, O75943, O43251 | | 2.28409091 |  |
| GO:0010605~negative regulation of macromolecule metabolic process | 3 | 0.49216694 | Q14865, O75943, O43251 | | 1.77163462 |  |
|  |  |  |  | |  |  |
| **Annotation Cluster 9** | **Enrichment Score: 0.24723493395354545** | | | | |  |
| Term | Count | PValue | Genes | | Fold Enrichment |  |
| GO:0046872~metal ion binding | 9 | 0.55166705 | Q96JW4, P62760, Q96KK3, Q9Y5Z6, Q86UR5, Q86W11, Q86XN6, Q9UBC2, Q7Z6K1 | | 1.1004444 |  |
| GO:0043169~cation binding | 9 | 0.56455457 | Q96JW4, P62760, Q96KK3, Q9Y5Z6, Q86UR5, Q86W11, Q86XN6, Q9UBC2, Q7Z6K1 | | 1.09000705 |  |
| GO:0043167~ion binding | 9 | 0.58198559 | Q96JW4, P62760, Q96KK3, Q9Y5Z6, Q86UR5, Q86W11, Q86XN6, Q9UBC2, Q7Z6K1 | | 1.07605877 |  |
|  |  |  |  | |  |  |
| **Annotation Cluster 10** | **Enrichment Score: 0.15894211366707897** | | | | |  |
| Term | Count | PValue | Genes | | Fold Enrichment |  |
| GO:0005524~ATP binding | 4 | 0.61259062 | Q99996, O94921, Q00532, O75943 | | 1.24476661 |  |
| GO:0032559~adenyl ribonucleotide binding | 4 | 0.62067815 | Q99996, O94921, Q00532, O75943 | | 1.23076923 |  |
| atp-binding | 4 | 0.64052746 | Q99996, O94921, Q00532, O75943 | | 1.20294887 |  |
| GO:0030554~adenyl nucleotide binding | 4 | 0.65793241 | Q99996, O94921, Q00532, O75943 | | 1.16791745 |  |
| GO:0001883~purine nucleoside binding | 4 | 0.66910535 | Q99996, O94921, Q00532, O75943 | | 1.14951815 |  |
| GO:0001882~nucleoside binding | 4 | 0.6743401 | Q99996, O94921, Q00532, O75943 | | 1.14095882 |  |
| GO:0032555~purine ribonucleotide binding | 4 | 0.75650368 | Q99996, O94921, Q00532, O75943 | | 1.01009077 |  |
| GO:0032553~ribonucleotide binding | 4 | 0.75650368 | Q99996, O94921, Q00532, O75943 | | 1.01009077 |  |
| GO:0017076~purine nucleotide binding | 4 | 0.78446782 | Q99996, O94921, Q00532, O75943 | | 0.96614609 |  |
| nucleotide-binding | 4 | 0.79296189 | Q99996, O94921, Q00532, O75943 | | 0.95055206 |  |
|  |  |  |  | |  |  |
| **Annotation Cluster 11** | **Enrichment Score: 0.07711546428640298** | | | | |  |
| Term | Count | PValue | Genes | | Fold Enrichment |  |
| zinc-finger | 4 | 0.70462785 | Q86UR5, Q86W11, Q86XN6, Q7Z6K1 | | 1.09457509 |  |
| GO:0008270~zinc ion binding | 4 | 0.8303973 | Q86UR5, Q86W11, Q86XN6, Q7Z6K1 | | 0.8929532 |  |
| zinc | 4 | 0.86628803 | Q86UR5, Q86W11, Q86XN6, Q7Z6K1 | | 0.82702735 |  |
| metal-binding | 4 | 0.9696837 | Q86UR5, Q86W11, Q86XN6, Q7Z6K1 | | 0.59584315 |  |
|  |  |  |  | |  |  |
| **Annotation Cluster 12** | **Enrichment Score: 0.04009090893199253** | | | | |  |
| Term | Count | PValue | Genes | | Fold Enrichment |  |
| GO:0006355~regulation of transcription, DNA-dependent | 3 | 0.87139231 | Q86W11, Q86XN6, Q14865 | | 0.85038462 |  |
| GO:0051252~regulation of RNA metabolic process | 3 | 0.88110162 | Q86W11, Q86XN6, Q14865 | | 0.82871064 |  |
| GO:0006350~transcription | 3 | 0.92546386 | Q86W11, Q86XN6, Q14865 | | 0.72254902 |  |
| transcription regulation | 3 | 0.93867719 | Q86W11, Q86XN6, Q14865 | | 0.68048686 |  |
| Transcription | 3 | 0.94499607 | Q86W11, Q86XN6, Q14865 | | 0.66205888 |  |
|  |  |  |  | |  |  |
|  |  |  |  | |  |  |

| **Brayton Point** |  |  |  |  |
| --- | --- | --- | --- | --- |
| **Annotation Cluster 1** | **Enrichment Score: 1.3429544851736586** | | | |
| Term | Count | PValue | Genes | Fold Enrichment |
| GTPase activation | 3 | 0.01451327 | P52757, Q9P2F8, Q9NZN5 | 15.3645985 |
| GO:0005096~GTPase activator activity | 3 | 0.03104939 | P52757, Q9P2F8, Q9NZN5 | 10.0403226 |
| GO:0030695~GTPase regulator activity | 3 | 0.09569893 | P52757, Q9P2F8, Q9NZN5 | 5.33571429 |
| GO:0060589~nucleoside-triphosphatase regulator activity | 3 | 0.0985046 | P52757, Q9P2F8, Q9NZN5 | 5.24578652 |
|  |  |  |  |  |
| **Annotation Cluster 2** | **Enrichment Score: 1.0203818845013437** | | | |
| Term | Count | PValue | Genes | Fold Enrichment |
| GO:0000902~cell morphogenesis | 3 | 0.08050559 | Q8TDC3, Q86WK6, P60880 | 5.93102127 |
| GO:0032989~cellular component morphogenesis | 3 | 0.09495459 | Q8TDC3, Q86WK6, P60880 | 5.38705186 |
| GO:0030182~neuron differentiation | 3 | 0.11363506 | Q8TDC3, Q86WK6, P60880 | 4.84261577 |
|  |  |  |  |  |
| **Annotation Cluster 3** | **Enrichment Score: 0.8155144601342057** | | | |
| Term | Count | PValue | Genes | Fold Enrichment |
| GO:0006917~induction of apoptosis | 3 | 0.06266038 | P62258, Q9NZN5, Q9C000 | 6.84520124 |
| GO:0012502~induction of programmed cell death | 3 | 0.06266038 | P62258, Q9NZN5, Q9C000 | 6.84520124 |
| GO:0043065~positive regulation of apoptosis | 3 | 0.10515556 | P62258, Q9NZN5, Q9C000 | 5.07193184 |
| GO:0043068~positive regulation of programmed cell death | 3 | 0.10564904 | P62258, Q9NZN5, Q9C000 | 5.05784314 |
| GO:0010942~positive regulation of cell death | 3 | 0.10663803 | P62258, Q9NZN5, Q9C000 | 5.02989925 |
| GO:0006915~apoptosis | 3 | 0.1712234 | P62258, Q9NZN5, Q9C000 | 3.76203209 |
| GO:0012501~programmed cell death | 3 | 0.17513259 | P62258, Q9NZN5, Q9C000 | 3.70839823 |
| GO:0008219~cell death | 3 | 0.23188653 | P62258, Q9NZN5, Q9C000 | 3.08614158 |
| GO:0016265~death | 3 | 0.23363823 | P62258, Q9NZN5, Q9C000 | 3.07052872 |
| GO:0042981~regulation of apoptosis | 3 | 0.27596694 | P62258, Q9NZN5, Q9C000 | 2.7380805 |
| GO:0043067~regulation of programmed cell death | 3 | 0.27950942 | P62258, Q9NZN5, Q9C000 | 2.71359691 |
| GO:0010941~regulation of cell death | 3 | 0.28069044 | P62258, Q9NZN5, Q9C000 | 2.70553273 |
|  |  |  |  |  |
| **Annotation Cluster 4** | **Enrichment Score: 0.4759114920799197** | | | |
| Term | Count | PValue | Genes | Fold Enrichment |
| GO:0005887~integral to plasma membrane | 3 | 0.2571884 | Q92859, P46093, P60880 | 2.79905437 |
| GO:0031226~intrinsic to plasma membrane | 3 | 0.2665904 | Q92859, P46093, P60880 | 2.73125721 |
| cell membrane | 3 | 0.54471537 | Q92859, P46093, P60880 | 1.59586808 |
|  |  |  |  |  |
| **Annotation Cluster 5** | **Enrichment Score: 0.39245203228056286** | | | |
| Term | Count | PValue | Genes | Fold Enrichment |
| zinc-finger | 4 | 0.22594571 | O00482, Q5VZL5, Q8TEJ3, P52757 | 2.2986077 |
| zinc | 4 | 0.37695143 | O00482, Q5VZL5, Q8TEJ3, P52757 | 1.73675743 |
| GO:0008270~zinc ion binding | 4 | 0.49160051 | O00482, Q5VZL5, Q8TEJ3, P52757 | 1.45104895 |
| GO:0046914~transition metal ion binding | 4 | 0.64311709 | O00482, Q5VZL5, Q8TEJ3, P52757 | 1.18346008 |
|  |  |  |  |  |
| **Annotation Cluster 6** | **Enrichment Score: 0.28854784810605655** | | | |
| Term | Count | PValue | Genes | Fold Enrichment |
| disulfide bond | 4 | 0.41990844 | Q92859, P46093, Q86WK6, P19835 | 1.62496984 |
| disulfide bond | 4 | 0.42597778 | Q92859, P46093, Q86WK6, P19835 | 1.61021228 |
| glycoprotein | 4 | 0.76175428 | Q92859, P46093, Q86WK6, P19835 | 1.00235714 |
|  |  |  |  |  |
| **Annotation Cluster 7** | **Enrichment Score: 0.25848790277245093** | | | |
| Term | Count | PValue | Genes | Fold Enrichment |
| GO:0031981~nuclear lumen | 3 | 0.45588117 | Q8TDC3, O43592, A7E2D6 | 1.82857143 |
| GO:0070013~intracellular organelle lumen | 3 | 0.57591927 | Q8TDC3, O43592, A7E2D6 | 1.48370927 |
| GO:0043233~organelle lumen | 3 | 0.58772238 | Q8TDC3, O43592, A7E2D6 | 1.45454545 |
| GO:0031974~membrane-enclosed lumen | 3 | 0.59932569 | Q8TDC3, O43592, A7E2D6 | 1.42650602 |
|  |  |  |  |  |
| **Annotation Cluster 8** | **Enrichment Score: 0.23060486591733503** | | | |
| Term | Count | PValue | Genes | Fold Enrichment |
| nucleotide phosphate-binding region:ATP | 3 | 0.31377542 | Q8TDC3, A7E2D6, Q9C000 | 2.51951372 |
| atp-binding | 3 | 0.45062337 | Q8TDC3, A7E2D6, Q9C000 | 1.89464446 |
| GO:0005524~ATP binding | 3 | 0.56955958 | Q8TDC3, A7E2D6, Q9C000 | 1.5170593 |
| GO:0032559~adenyl ribonucleotide binding | 3 | 0.57609662 | Q8TDC3, A7E2D6, Q9C000 | 1.5 |
| nucleotide-binding | 3 | 0.58099459 | Q8TDC3, A7E2D6, Q9C000 | 1.49711949 |
| GO:0030554~adenyl nucleotide binding | 3 | 0.6065052 | Q8TDC3, A7E2D6, Q9C000 | 1.42339939 |
| GO:0001883~purine nucleoside binding | 3 | 0.61573417 | Q8TDC3, A7E2D6, Q9C000 | 1.40097524 |
| GO:0001882~nucleoside binding | 3 | 0.62007776 | Q8TDC3, A7E2D6, Q9C000 | 1.39054356 |
| GO:0032553~ribonucleotide binding | 3 | 0.69033364 | Q8TDC3, A7E2D6, Q9C000 | 1.23104812 |
| GO:0032555~purine ribonucleotide binding | 3 | 0.69033364 | Q8TDC3, A7E2D6, Q9C000 | 1.23104812 |
| GO:0017076~purine nucleotide binding | 3 | 0.71541571 | Q8TDC3, A7E2D6, Q9C000 | 1.17749054 |
| GO:0000166~nucleotide binding | 3 | 0.80306726 | Q8TDC3, A7E2D6, Q9C000 | 0.99812934 |
|  |  |  |  |  |
| **Annotation Cluster 9** | **Enrichment Score: 0.20582001068436045** | | | |
| Term | Count | PValue | Genes | Fold Enrichment |
| dna-binding | 3 | 0.55870205 | O00482, Q96I24, Q8WY36 | 1.55691568 |
| transcription regulation | 3 | 0.60760719 | O00482, Q96I24, Q8WY36 | 1.4290224 |
| Transcription | 3 | 0.62331294 | O00482, Q96I24, Q8WY36 | 1.39032365 |
| GO:0006350~transcription | 3 | 0.70992201 | O00482, Q96I24, Q8WY36 | 1.19008074 |
|  |  |  |  |  |
| **Annotation Cluster 10** | **Enrichment Score: 0.2030650519571948** | | | |
| Term | Count | PValue | Genes | Fold Enrichment |
| metal-binding | 5 | 0.36121042 | O00482, Q5VZL5, Q8TEJ3, Q8TDC3, P52757 | 1.56408827 |
| GO:0046872~metal ion binding | 5 | 0.74396216 | O00482, Q5VZL5, Q8TEJ3, Q8TDC3, P52757 | 0.99345675 |
| GO:0043169~cation binding | 5 | 0.7519173 | O00482, Q5VZL5, Q8TEJ3, Q8TDC3, P52757 | 0.98403414 |
| GO:0043167~ion binding | 5 | 0.76253293 | O00482, Q5VZL5, Q8TEJ3, Q8TDC3, P52757 | 0.97144195 |
|  |  |  |  |  |
| **Annotation Cluster 11** | **Enrichment Score: 0.06273936436718693** | | | |
| Term | Count | PValue | Genes | Fold Enrichment |
| topological domain:Extracellular | 3 | 0.6859416 | Q92859, P46093, Q86WK6 | 1.24500924 |
| topological domain:Cytoplasmic | 3 | 0.83043597 | Q92859, P46093, Q86WK6 | 0.94071229 |
| glycosylation site:N-linked (GlcNAc...) | 3 | 0.91353185 | Q92859, P46093, Q86WK6 | 0.75907213 |
| transmembrane | 3 | 0.96580664 | Q92859, P46093, Q86WK6 | 0.61190407 |
| transmembrane region | 3 | 0.96626446 | Q92859, P46093, Q86WK6 | 0.6102839 |
